# The centrosome-cilium-centriolar satellite axis in neurodegenerative diseases

**DOI:** 10.1038/s44319-026-00877-3

**Published:** 2026-07-21

**Authors:** Umut Sahin, Elif Nur Firat-Karalar

**Affiliations:** 1 Faculty of Engineering and Natural Sciences, Sabancı University, Istanbul, Türkiye; 2 Department of Molecular Biology and Genetics, Koç University, Istanbul, Türkiye; 3 School of Medicine, Koç University, Istanbul, Türkiye

**Keywords:** Molecular Biology of Disease, Organelles, Signal Transduction

## Abstract

Neurodegenerative diseases (NDDs), including Alzheimer’s disease, Parkinson’s disease, Huntington’s disease, amyotrophic lateral sclerosis, and hereditary ataxias, remain major global health challenges with limited therapeutic options. Although clinically and genetically diverse, these diseases share extensively studied cellular and molecular hallmarks, including protein aggregation, impaired proteostasis, cytoskeletal abnormalities, altered energy metabolism, nucleic acid damage, and chronic inflammation. Emerging evidence indicates that dysfunction of the centrosome–cilium–satellite axis intersects with these established pathways in disease- and cell type-specific contexts. This axis, composed of centrosomes, primary cilia, and centriolar satellites, coordinates cytoskeletal organization, ciliary signaling, trafficking, proteostasis, and stress responses and acquires specialized functions in neurons that support polarity, connectivity, and long-term maintenance. In this review, we outline the structure, function, and neuronal specializations of the centrosome–cilium–satellite axis, then examine how its dysfunction has been reported in neurodegenerative disease models. We also discuss centriolar satellites as regulators of centrosome and cilium biology whose disease-specific roles in classical NDDs remain comparatively underexplored, with insights from Huntington’s disease and schizophrenia. Finally, we discuss therapeutic strategies aimed at restoring axis structure and dynamics, modulating ciliary signaling, and correcting disease-linked genetic or transcript-level defects, emphasizing mechanism-based approaches that require validation in disease-relevant models. Together, the centrosome–cilium–satellite axis provides an emerging framework for understanding context-dependent organelle dysfunction in neuronal vulnerability and neurodegeneration.

## Abstract (web summary)

Emerging evidence indicates a role for the centrosome-cilium-satellite axis in neurodegenerative diseases. This review describes the structure and neuronal adaptations of this axis, evaluates alterations in neurodegenerative disease, and discusses potential mechanism-based therapeutic strategies.

## Graphical abstract

**Figure:**
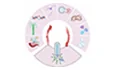

## Classical hallmarks of neurodegenerative diseases

Neurodegenerative diseases (NDDs) are a heterogeneous group of conditions characterized by progressive loss of neurons in the central nervous system and, in some cases, the peripheral nervous system. With a globally aging population and limited therapeutic options, their burden continues to rise, representing one of the most pressing public health challenges worldwide. Common NDDs include Alzheimer’s disease (AD), Parkinson’s disease (PD), amyotrophic lateral sclerosis (ALS), Huntington’s disease (HD), and hereditary ataxias, among others (Gadhave et al, 2024; Habib et al, 2018; Wilson et al, 2023). Each disorder is defined by distinct combinations of cognitive, motor, and neuropsychiatric, and systemic manifestations, reflecting substantial clinical, genetic, and pathological heterogeneity. A systems-level understanding of the mechanisms driving disease onset and progression is therefore essential for developing effective and disease-specific interventions. Achieving this remains challenging as NDDs are complex and multifactorial disorders in which different pathogenic mechanisms may dominate depending on disease type, genetic background, affected cell population, and disease stage.

Despite this diversity, decades of research have identified a shared set of cellular and molecular hallmarks that provide the major conceptual framework for understanding neurodegeneration (Wilson et al, 2023). These include pathological protein aggregation, impaired proteostasis, cytoskeletal and axonal transport abnormalities, altered energy metabolism and mitochondrial dysfunction, DNA and RNA defects, lysosomal-autophagic impairment, and chronic inflammation, ultimately leading to synaptic and network failure and neuronal death. The causal status of these hallmarks varies across diseases and evidence types. In some contexts, disease-causing mutations or pathological protein accumulation directly implicate specific pathways in disease onset or progression; in others, the same cellular processes may act as downstream consequences, modifiers, or amplifiers of neuronal vulnerability. This variability is central to understanding why NDDs share molecular features while remaining clinically and mechanistically distinct. Because these hallmarks have been extensively reviewed elsewhere, we do not discuss each of them in detail here; instead, we use them as an established framework for evaluating how additional organelle-level phenotypes intersect with neurodegenerative mechanisms.

These hallmarks are interconnected rather than independent endpoints. Proteostasis failure can impair mitochondrial function, cytoskeletal disruption can compromise axonal transport and synaptic maintenance, inflammatory signaling can exacerbate oxidative and metabolic stress, and nucleic acid damage can further destabilize neuronal homeostasis. Such reciprocal interactions contribute to the progressive and cell-type-selective nature of neurodegeneration. Moreover, the heterogeneity seen within and across NDDs indicates that established hallmarks do not fully capture all aspects of disease complexity. This has motivated increasing interest in additional organelle-level pathways that may intersect with classical mechanisms. In this context, centrosomes, primary cilia, and centriolar satellites have emerged as cellular structures of interest because they regulate cytoskeletal organization, polarized trafficking, signal transduction, proteostasis, stress responses, and neuronal homeostasis (Lattao, 2025; Takahashi et al, 2025).

## The centrosome–cilium–satellite axis in neuronal homeostasis and vulnerability

Centrosomes, primary cilia, and centriolar satellites form a spatially and functionally connected axis that supports neuronal homeostasis. The centrosome organizes microtubules; in ciliated cells, the mother centriole can serve as the basal body during ciliogenesis; primary cilia transduce developmental and homeostatic signaling pathways; and centriolar satellites regulate the trafficking, composition, and remodeling of centrosomal and ciliary proteins (Begar et al, 2024; Devi et al, 2021). Historically, centrosomes and primary cilia were studied mainly in the context of cell division and developmental signaling, with their dysfunction linked to ciliopathies, neurodevelopmental disorders, and microcephaly (Nigg and Raff, 2009). More recently, centriolar satellites have emerged as dynamic regulators of centrosome and cilium assembly, signaling, proteostasis, autophagy, and stress responses (Begar et al, 2024). Together, these structures coordinate processes that are particularly important in neurons, including polarity, polarized trafficking, signaling, protein quality control, and adaptation to cellular stress.

In this review, we examine reported organelle-level alterations affecting the centrosome–cilium–satellite axis in NDD models, while noting that the strength of evidence varies across diseases and axis components. Centrosomal and ciliary defects have been more broadly documented across NDD models, whereas centriolar satellite-specific mechanisms remain less extensively characterized in classical NDDs. In the sections that follow, we first introduce the structure, function, biogenesis, and neuronal adaptations of the centrosome–cilium–satellite axis. We then discuss ciliopathies as reference models for centrosome–cilium dysfunction in the nervous system. The disease-focused sections evaluate reported axis alterations in major NDDs and in schizophrenia as a related neurodevelopmental and neuropsychiatric context, emphasizing how these phenotypes intersect with established disease mechanisms. We close by discussing therapeutic opportunities and key open questions regarding timing, causality, cell-type specificity, and disease-stage dependence.

### The structure, function, and biogenesis of the centrosome–cilium–satellite axis

**Figure 1:**
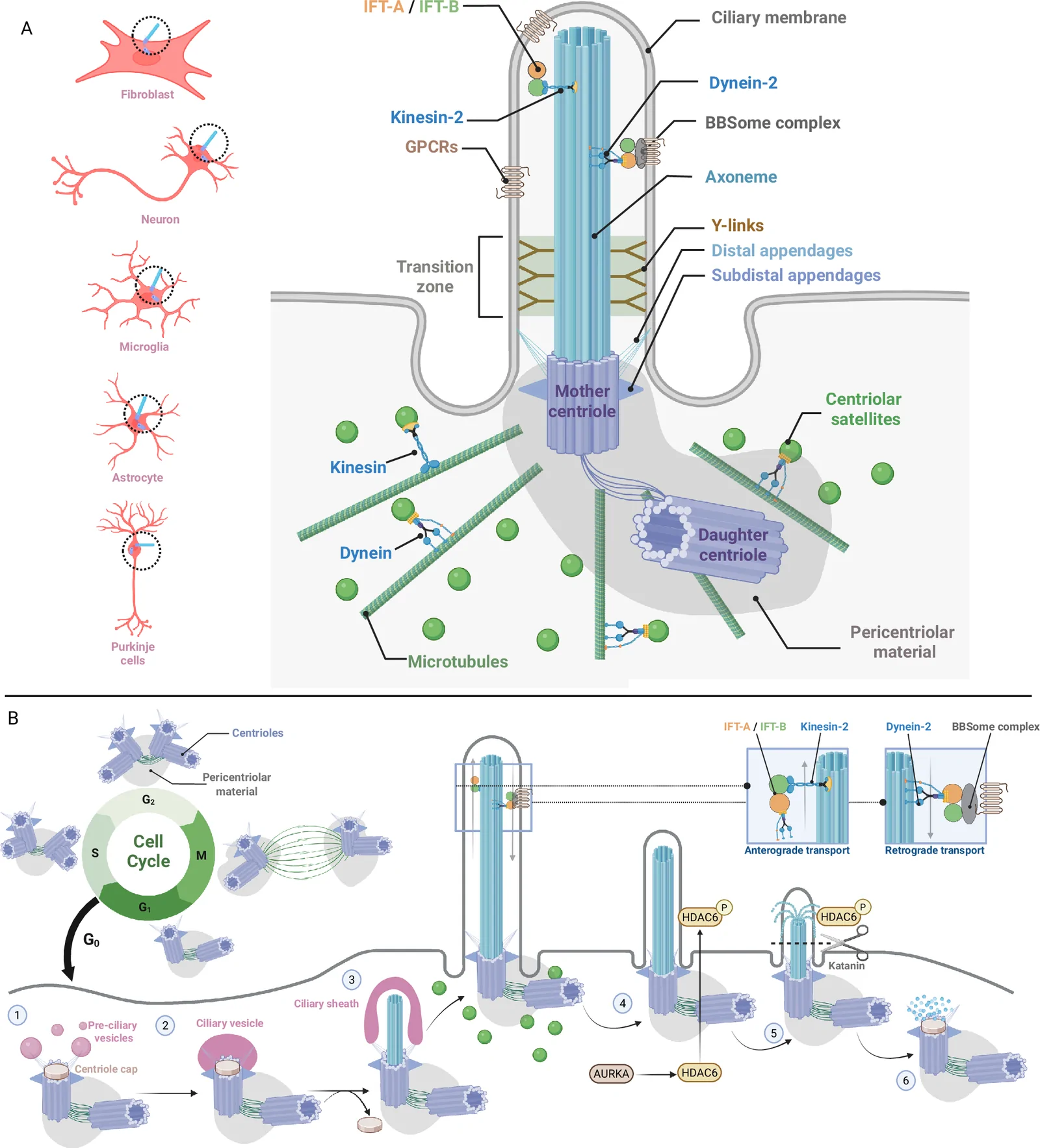
Structure and biogenesis of the centrosome–cilium–satellite axis. (**A**) Fibroblasts, neurons, microglia, astrocytes, and Purkinje cells are shown with a primary cilium, and the zoomed-in panel depicts the major components of the centrosome–cilium–satellite axis. The centrosome consists of a mother and a daughter centriole surrounded by pericentriolar material (PCM) that nucleates and organizes microtubules. The mother centriole contains distal and subdistal appendages; distal appendages mediate docking to the plasma membrane in cells that form a primary cilium, whereas subdistal appendages contribute to microtubule anchoring. When the mother centriole docks at the plasma membrane, it functions as the basal body that nucleates axoneme assembly. During ciliogenesis, the distal appendages of the mother centriole function as transition fibers that anchor the basal body to the membrane. The primary cilium comprises the axoneme enclosed by a specialized ciliary membrane enriched in signaling receptors such as GPCRs. Axonemal transport is driven by intraflagellar transport (IFT), with IFT-B/kinesin-2 mediating anterograde transport and IFT-A/dynein-2 supporting retrograde transport. The transition zone forms a selective barrier that regulates ciliary composition, with Y-links connecting the axoneme to the ciliary membrane. The BBSome cooperates with the IFT machinery to sort and retrieve GPCRs and other membrane proteins. Centriolar satellites, scaffolded by PCM1, are membrane-less granules that cluster around centrosomes and basal bodies to regulate trafficking, ciliogenesis, ciliary signaling, proteostasis, and stress responses. (**B**) Biogenesis of the centrosome–cilium–satellite axis. Each centriole duplicates once at the G1/S transition and elongates through S-G2, and centriole pairs are segregated to daughter cells during mitosis. Centrosomes mature in late G2-M through expansion of the PCM. Upon cell-cycle exit or differentiation, the mother centriole becomes the basal body and initiates ciliogenesis. (1) Pre-ciliary vesicles are recruited to distal appendages through membrane-trafficking pathways involving Rab GTPases, while the CP110–CEP97 cap remains at the distal end of the mother centriole to suppress premature axoneme extension. (2) These vesicles fuse to form the ciliary vesicle. (3) Recruitment of TTBK2 promotes the removal of the CP110–CEP97 cap and recruitment of IFT machinery, enabling axoneme initiation. The ciliary sheath expands into the mature ciliary membrane through BBSome-dependent and small GTPase-regulated trafficking. (4–6) Before mitotic re-entry or in response to extracellular cues, the cilium disassembles through mechanisms that include AURKA-HDAC6-mediated axonemal destabilization, ciliary resorption, and katanin-dependent scission. Centriolar satellites undergo parallel cycles of assembly, disassembly, and remodeling, enabling context-dependent regulation of centrosome and cilium composition. Created in BioRender. Begar, E. (2026) https://BioRender.com/qit92k3.

The centrosome, primary cilium, and centriolar satellites form a spatially and functionally connected axis that regulates processes essential for development and physiology (Fig. 1A) (Begar et al, 2024; Chavali et al, 2014; Werner et al, 2017). Mutations in genes encoding centrosome, cilium, or centriolar satellite proteins cause a spectrum of inherited disorders known as ciliopathies (Mill et al, 2023; Reiter and Leroux, 2017). These multisystemic diseases manifest with diverse clinical features, including retinal degeneration, skeletal abnormalities, obesity, and polycystic kidney disease. Some are relatively organ-specific, such as polycystic kidney disease, which affects the kidney, whereas others, including Joubert and Bardet–Biedl syndromes, involve multiple organ systems with prominent neurological symptoms. Consistent with the tissue-specific manifestations of ciliopathies, the architecture, functions, and biogenesis of this evolutionarily conserved axis exhibit cell type-dependent variation in mammals. The following section outlines the conserved structural and functional features of its components, mechanisms governing their assembly and disassembly, and neuron-specific adaptations that underpin brain development and function. Together, these provide the conceptual foundation for interpreting centrosome-, cilium-, and satellite-associated phenotypes reported in NDD models.

### Core structure and functions of the centrosome–cilium–satellite axis

The centrosome is the major microtubule-organizing center (MTOC) in most animal cells (Fig. 1A) (Conduit et al, 2015; Petry and Vale, 2015). During interphase, it nucleates and anchors microtubules to control cell shape, motility, polarity, and intracellular transport. In mitosis, duplicated centrosomes migrate to opposite poles and organize the bipolar spindle to ensure accurate chromosome segregation. Beyond its MTOC role, the centrosome also acts as a signaling hub by concentrating regulatory molecules involved in processes such as cell cycle control, DNA damage response, and other pathways (Arquint et al, 2014). Structurally, the centrosome consists of two centrioles surrounded by the pericentriolar material (PCM), which contains microtubule-nucleating factors such as the gamma-tubulin ring complex that confer the centrosome’s MTOC activity. Centrioles are microtubule-based cylindrical structures that are structurally and functionally distinct. The mother centriole bears distal and subdistal appendages, whereas the daughter centriole lacks these appendages until maturation (Kumar and Reiter, 2021). Centrioles are required to assemble centrosomes by recruiting PCM and, in cells that exit the cell cycle, the mother centriole docks at the plasma membrane through its distal appendages and functions as the basal body that templates primary cilium formation (Gupta and Kitagawa, 2018; Loncarek and Bettencourt-Dias, 2018).

The primary cilium is a non-motile, antenna-like projection that acts as a sensory hub for diverse signaling pathways, including Sonic Hedgehog (Shh), Wnt, and platelet-derived growth factor (PDGF) (Fig. 1A) (Brown and Zhang, 2020; Mill et al, 2023; Nishimura et al, 2019). At its core lies the axoneme, a microtubule-based scaffold whose proper length and structural stability are crucial for ciliary function (Deretic et al, 2023; Pedersen et al, 2012). The axoneme is enclosed by a specialized ciliary membrane that is enriched in signaling receptors such as G protein-coupled receptors (GPCRs), ion channels, and components of the cAMP and Ca²⁺ signaling pathways. The assembly, localization, and turnover of these components depend on intraflagellar transport (IFT), which moves cargo bidirectionally along the axoneme to assemble, maintain, and remodel the cilium (Nachury, 2018; Nachury and Mick, 2019). The BBSome complex cooperates with the IFT machinery to mediate selective trafficking and retrieval of membrane proteins within the cilium (Tian et al, 2023). Tight regulation of ciliary structure, signaling, composition, and function is essential, as their disruption leads to ciliopathies affecting the kidney, retina, brain, and skeleton (Braun and Hildebrandt, 2017; Reiter and Leroux, 2017). Although this review focuses mainly on primary cilia, motile cilia of ependymal cells are also relevant to brain homeostasis because they regulate cerebrospinal fluid flow (Ma et al, 2023b; Nelles and Hazrati, 2022).

Centriolar satellites (hereafter, satellites), previously regarded as accessory structures, are now recognized as dynamic components that function within the centrosome–cilium–satellite axis (Fig. 1A) (Begar et al, 2024; Prosser and Pelletier, 2020). These small (70–100 nm) membrane-less granules, scaffolded by the Pericentriolar Material 1 (PCM1) protein, typically cluster around the centrosome in a microtubule- and motor-dependent manner (Kubo et al, 1999). Satellite number, distribution, size, and composition vary across cellular contexts; for example, satellites form fibrous granules in multiciliated cells and show distinct organization in differentiated cell types, including neurons. Quantitative mass spectrometry of PCM1-purified satellites and proximity-labeling approaches such as BioID have expanded the satellite proteome and interactome, identifying more than 200 satellite-associated proteins enriched in centrosomal and ciliary proteins, microtubule-associated proteins, RNA-binding proteins, and quality-control factors; additionally, satellites also contain mRNAs (Gheiratmand et al, 2019; Pachinger et al, 2025; Quarantotti et al, 2019). Consistent with this composition, satellites act as multifunctional sites where proteins can be assembled into complexes, stored, post-translationally modified, locally translated, or trafficked to centrosomes and cilia. Their best-characterized functions center on the primary cilium, where they regulate ciliogenesis and control trafficking of structural and signaling components required for pathways such as Shh signaling. These ciliary roles are cell-type-dependent, as satellites are required for efficient ciliogenesis in some epithelial contexts but are partially dispensable in others (Hall et al, 2023; Hoang-Minh et al, 2016; Odabasi et al, 2019; Zhao et al, 2021). Autophagy further links satellites to ciliogenesis and centrosome–cilium homeostasis in a cargo- and context-dependent manner. Selective autophagic degradation of the satellite-localized pool of OFD1 promotes ciliogenesis, whereas other autophagy-dependent mechanisms, such as degradation of IFT20 in cycling cells, can restrict cilium formation (Pampliega et al, 2013; Tang et al, 2013). Beyond their ciliary roles, satellites contribute to proteostasis and stress responses by regulating protein stability through the ubiquitin-proteasome system and autophagy, and by promoting aggresome formation to sequester misfolded or aggregated proteins for degradation (Begar et al, 2024; Holdgaard et al, 2019; Joachim et al, 2017; Lecland and Merdes, 2018; Prosser et al, 2022; Villumsen et al, 2013). Consistent with their ciliary functions, Pcm1 knockout animal models exhibit distinct developmental and neuronal phenotypes relevant to ciliopathies and schizophrenia (Hall et al, 2023; Huang et al, 2025b; Monroe et al, 2020). Together, these findings define satellites as regulators of ciliary trafficking, ciliogenesis, autophagy, proteostasis, and centrosome integrity. Their direct disease-specific roles in classical NDDs remain less extensively characterized than those of centrosomes and primary cilia, but their established functions in centrosome–cilium regulation make them important components of the axis considered in this review.

### Biogenesis of the centrosomes, primary cilium, and centriolar satellites

Biogenesis of the centrosome–cilium–satellite axis is tightly coordinated with the cell cycle and cellular state (Fig. 1B). At the G1/S transition, new centrioles begin to form perpendicular to each pre-existing (mother) centriole in a process known as centriole duplication, which is tightly regulated to occur once and only once per cell cycle in most cells (Breslow and Holland, 2019; Loncarek and Bettencourt-Dias, 2018). The newly formed daughter centrioles elongate during the S and G2 phases, and each centriole pair is subsequently segregated equally to daughter cells, ensuring faithful centrosome inheritance. Concurrently, in late G2 and mitosis, the centrosome undergoes maturation characterized by expansion of the pericentriolar material (PCM) and increased microtubule nucleation activity (Blanco-Ameijeiras et al, 2022; Palazzo et al, 2000). After mitotic exit, the newly formed daughter centrioles complete centriole-to-centrosome conversion and acquire distal and subdistal appendages, enabling them to function as mature mother centrioles in the next cell cycle (Blanco-Ameijeiras et al, 2022).

When cells exit the cell cycle and enter quiescence or differentiation, the mother centriole shifts from its role within the centrosome to function as the basal body that nucleates and organizes primary cilium formation (Breslow and Holland, 2019; Mirvis et al, 2018). Ciliogenesis is a multistep, highly coordinated process that requires the interplay of numerous proteins, pathways, and organelles (Fig. 1B). In the intracellular ciliogenesis pathway, pre-ciliary vesicles are recruited to the distal appendages of the mother centriole, where they fuse to form a ciliary vesicle that caps the distal end of the centriole. Distal appendage proteins such as CEP164, CEP83, and SCLT1 support this early docking and vesicle recruitment step through small GTPases, including RAB8 and RAB11, while transition-zone components (e.g., CEP290, NPHP, and TMEM family proteins) establish a selective barrier at the ciliary base (Goncalves and Pelletier, 2017; Kumar and Reiter, 2021; Ma et al, 2023a). In proliferating cells, axoneme extension is suppressed by the CP110–CEP97 complex, which forms an inhibitory cap at the distal end of the mother centriole (Spektor et al, 2007). Removal of this cap, triggered by the recruitment of the kinase TTBK2 to distal appendages and coordinated with the action of CEP164 and CEP83, marks the initiation of ciliogenesis (Loukil et al, 2021; Tanos et al, 2013). As the axoneme begins to elongate, the ciliary vesicle expands around the growing axoneme to form a ciliary sheath, which subsequently fuses with the plasma membrane to expose the cilium at the cell surface. Further axoneme elongation and ciliary maintenance depend on the IFT machinery, composed of IFT-A and IFT-B complexes and their associated motors kinesin-2 and dynein-2, as well as small GTPases that regulate ciliary membrane trafficking (Blacque et al, 2018; Ishikawa and Marshall, 2017). Once assembled, the cilium maintains its length and architecture through continuous IFT-mediated turnover and dynamic remodeling in response to developmental and environmental cues (Werner et al, 2017). Before mitotic re-entry or in response to extracellular stimuli, the primary cilium disassembles via AURKA-HDAC6-mediated axonemal destabilization, katanin-dependent scission, or a combination of both (Fig. 1B) (Liang et al, 2016; Mirvis et al, 2018). Recent studies further show that in differentiating cells, such as adipocytes and neurons, cilia disassemble through alternative, context-dependent pathways (Ott et al, 2024a; Ott and Mukhopadhyay, 2025).

In parallel with the dynamic events of centriole and cilium biogenesis during the cell cycle, centriolar satellites also undergo regulated cycles of assembly, disassembly, and remodeling. Satellite assembly is initiated by the formation of a PCM1 scaffold, which then hierarchically recruits other satellite proteins, including ciliopathy-linked proteins such as OFD1 and CEP131, to form granules that support centrosome and cilium function (Begar et al, 2026). In most interphase cells, satellites are concentrated around the centrosome or basal body, with a subset distributed more broadly throughout the cytoplasm. Upon mitotic entry, satellites disassemble in a kinase-dependent manner and subsequently reassemble following mitotic exit (Dammermann and Merdes, 2002; Rai et al, 2018). Beyond these cell cycle-linked transitions, satellites also remodel in different cellular contexts. Large fibrous granules form during differentiation to support centriole amplification and ciliogenesis in multiciliated epithelial cells, whereas in muscle cells, satellites cluster at the nuclear envelope (Kubo and Tsukita, 2003; Odabasi et al, 2020). These dynamic behaviors highlight the highly plastic nature of satellites, which enables them to adapt their homeostatic properties to both cellular state and cell type. This plasticity is important for interpreting disease phenotypes, because satellite organization and function may vary substantially across neural cell types and disease contexts.

### The neuronal adaptations of the centrosome–cilium–satellite axis

Neurons are highly polarized, post-mitotic cells whose axons and dendrites require specialized cytoskeletal organization and polarized trafficking (Florio and Huttner, 2014; Taverna et al, 2014). During development, newly differentiated neurons extend neurites that later specify axonal or dendritic identity, establishing the architecture required for neuronal connectivity (Akhmanova and Kapitein, 2022; Conde and Caceres, 2009; Kapitein and Hoogenraad, 2015). This architecture is built and maintained largely by the microtubule cytoskeleton. Axons contain predominantly uniform microtubule arrays with plus ends oriented away from the soma, whereas dendrites in vertebrates contain mixed-polarity arrays (Baas et al, 1988; Goodwin et al, 2012; Stone et al, 2008; Yau et al, 2016). In migrating newborn neurons, centrosomes act as the major MTOC. During maturation, the centrosome gradually loses its MTOC activity, and microtubule growth occurs at non-centrosomal sites such as the Golgi, endosomes, or pre-existing microtubules (Vinopal and Bradke, 2025). Despite this functional transition, mature neurons retain the centrosome as the basal body that anchors the primary cilium and as an organizer that supports polarized transport required for axonal and synaptic function (Jurisch-Yaksi et al, 2024). Recent spatial proteomic analyses of neural centrosomes revealed major compositional changes upon neuronal differentiation. Nearly 60% of centrosome-associated proteins in neural stem cells and neurons differ from those in other cell types, and neuronal centrosomes are notably enriched in RNA-binding and splicing factors, suggesting specialized roles in local RNA regulation (O’Neill et al, 2022). This neural remodeling may help explain how broadly expressed centrosomal proteins acquire cell-type-specific functions, and why mutations in centrosomal genes can produce selective neurological phenotypes.

Primary cilia in the nervous system are important sensory and signaling organelles that regulate neural development, differentiation, and long-term homeostasis. They mediate key pathways such as Shh and Wnt during neurogenesis and also modulate stem cell activity, regeneration, and neuronal signaling in the adult nervous system (Guemez-Gamboa et al, 2014). Many cell types in both the central and peripheral nervous systems, including most mature CNS neurons, sensory and motor neurons, and glial cells such as astrocytes, have a primary cilium, underscoring its broad yet specialized roles in neural function (Fig. 1A) (Louvi and Grove, 2011). The length, morphology, and receptor composition of neuronal cilia vary across cell types, brain regions, and activity states, reflecting cell type-specific signaling demands (Jurisch-Yaksi et al, 2024). During neurogenesis, cilia on neural progenitors and newborn neurons transduce morphogenetic and metabolic signals, most prominently through the Shh pathway, which regulates self-renewal, differentiation, migration, and synaptogenesis (Suciu and Caspary, 2021). As neurons mature, their cilia become specialized in structure and composition, reflecting the distinct signaling requirements of different neuronal subtypes. In mature neurons, cilia contain GPCRs for neuromodulators such as serotonin, dopamine, and somatostatin, as well as ion channels and signaling proteins that modulate excitability and synaptic plasticity (Hilgendorf et al, 2016; Loukil et al, 2025).

Neuronal cilia typically protrude from the soma or a proximal dendrite and are positioned near synapses, where they can sense locally released neurotransmitters and neuropeptides (Gopalakrishnan et al, 2023; Jurisch-Yaksi et al, 2024) (Jurisch-Yaksi et al, 2024; Ott et al, 2024b; Wu et al, 2024). In most neurons, chemical communication occurs through well-characterized synapses formed between the axon of one neuron and the dendrite of another. Recent studies have expanded this view by revealing that neuronal signaling can also occur through direct contacts between axons and cilia. A specialized axo-ciliary synapse between serotonergic axons and the primary cilia of pyramidal neurons in the hippocampal CA1 region demonstrates that cilia can act as postsynaptic signaling sites, coupling neurotransmitter release to transcriptional and metabolic responses in the receiving neuron (Sheu et al, 2022). This is supported by in vivo proximity and in situ proteomic studies, which revealed neuron-specific signaling networks enriched in synaptic, receptor, and adhesion components and identified discrete ciliary nanodomains positioned to sense neurotransmitter efflux (Chang et al, 2026; Loukil et al, 2025). Extending this view of cilia-synapse communication, time-resolved proximity proteomics of human hypothalamic neurons revealed ciliary enrichment of synaptic, receptor, and adhesion proteins (Macarelli et al, 2025). Moreover, proximity labeling and quantitative proteomics in the developing mouse brain mapped region-specific radial glial cilia proteomes, identified translation-related components, and implicated ciliary proteins in cortical development (Liu et al, 2025). Together, these findings highlight the functional versatility of neuronal cilia as integrators of synaptic, metabolic, and developmental signals, positioning them as key modulators of neuronal communication and circuit organization.

Centriolar satellites have recently emerged as important regulators of neuronal development and homeostasis, although their functions in the nervous system remain less extensively characterized than those of centrosomes and primary cilia. Characterization of two independent *Pcm1*^*−/−*^ mouse models, generated by gene-trap and CRISPR/Cas9 strategies, has provided complementary insights into satellite functions in the brain (Hall et al, 2023; Monroe et al, 2020). The gene-trap model exhibits behavioral and cognitive phenotypes relevant to schizophrenia, accompanied by progressive ciliary and anatomical abnormalities (Monroe et al, 2020). In contrast, the CRISPR/Cas9 model displays ciliopathy-like developmental defects, such as hydrocephalus, cerebellar hypoplasia, oligospermia, and progressive ciliary degeneration, reflecting a broader requirement for satellites in maintaining ciliary structure and signaling (Hall et al, 2023). Despite differences in severity and timing, both models point to ciliary dysfunction as a major consequence of impaired satellite function in the nervous system. Beyond ciliogenesis, satellites may also contribute to neuronal processes through their involvement in cellular stress responses and proteostatic regulation. They regulate protein stability via the ubiquitin-proteasome system and autophagy and promote aggresome formation to sequester misfolded or aggregated proteins for degradation (Joachim et al, 2017; Lecland and Merdes, 2018; Prosser et al, 2022; Villumsen et al, 2013; Wang et al, 2016). Although the precise contribution of this proteostatic function in neurons remains to be clarified, these findings suggest that satellites may support neuronal resilience by contributing to protein quality control, a process that declines with aging and is implicated in NDD vulnerability.

### Ciliopathies as models of centrosome–cilium–satellite dysfunction in the nervous system

Ciliopathies are rare inherited disorders caused by defective ciliogenesis, ciliary structure, trafficking, or signaling (Mill et al, 2023; Reiter and Leroux, 2017). Because cilia are broadly required for tissue development and homeostasis, ciliopathies typically affect multiple organ systems, including the kidney, retina, skeleton, liver, and brain. Their neurological manifestations provide an important reference point for understanding how centrosome- and cilium-dependent pathways contribute to nervous system development, tissue organization, and long-term cellular maintenance (Serpieri et al, 2025; Volos et al, 2025). For example, Joubert syndrome illustrates the neurodevelopmental impact of ciliary dysfunction, with characteristic cerebellar and brainstem malformations accompanied by hypotonia, ataxia, abnormal eye movements, respiratory dysregulation, and cognitive impairment (Gana et al, 2022; Parisi, 2019). Bardet–Biedl syndrome similarly highlights the neurological and sensory consequences of ciliary defects, including retinal cone-rod dystrophy, cognitive impairment, developmental delay, poor coordination, olfactory dysfunction, and behavioral abnormalities (Tian et al, 2023; Wang et al, 2021).

Ciliopathies also show that ciliary dysfunction can affect long-term maintenance of specialized neural cells, not only early development. Progressive retinal degeneration, a hallmark manifestation of many ciliopathies, is the clearest example (Bujakowska et al, 2017; May-Simera et al, 2017). Photoreceptor outer segments are highly specialized sensory cilia, and defective ciliary trafficking in retinal ciliopathies compromises photoreceptor function and survival over time. Motile cilia provide an additional link to nervous system homeostasis (Hyland and Brody, 2021; Wallmeier et al, 2020). Ependymal motile cilia lining the cerebral ventricles generate directional cerebrospinal fluid (CSF) flow, and their dysfunction can contribute to hydrocephalus and altered ventricular homeostasis. Because CSF circulation contributes to nutrient distribution, CSF-interstitial fluid exchange, and waste clearance, impaired ependymal ciliary function may also affect clearance pathways relevant to adult-onset NDD biology (Ma et al, 2023b; Nelles and Hazrati, 2022).

Animal, iPSC, and organoid models further support the relevance of centrosome–cilium biology to neural development and tissue organization. Mouse ciliopathy models have linked disrupted ciliary signaling to hydrocephalus, neural progenitor abnormalities, and altered brain structure (Nishimura et al, 2004; Norris and Grimes, 2012; Rachel et al, 2015; Zhang et al, 2011). Human iPSC-derived neural and brain organoid models are beginning to extend these observations to human cellular contexts, revealing ciliopathy-associated defects in ventricular architecture, neural differentiation, and early tissue organization (Eschment et al, 2025; Shaikh et al, 2025). These models are particularly useful for studying developmental and tissue-level consequences of ciliary dysfunction, although they do not yet fully capture the age-dependent progression, mature circuit dysfunction, or long-term degenerative features of adult-onset NDDs.

Together, ciliopathy studies support the biological relevance of centrosome–cilium dysfunction in the nervous system by showing how defects in ciliary assembly, trafficking, signaling, or motility can disrupt neural development, ventricular and retinal homeostasis, and selected neural cell populations. At the same time, ciliopathies and adult-onset NDDs remain distinct disease contexts. We therefore use ciliopathy biology to inform the interpretation of centrosome–cilium defects reported in NDD models, while evaluating NDD-associated evidence within its own genetic, cellular, pathological, and disease-stage-specific framework.

## Centrosome–cilium–satellite axis alterations in NDDs and related neurodevelopmental contexts

**Figure 2:**
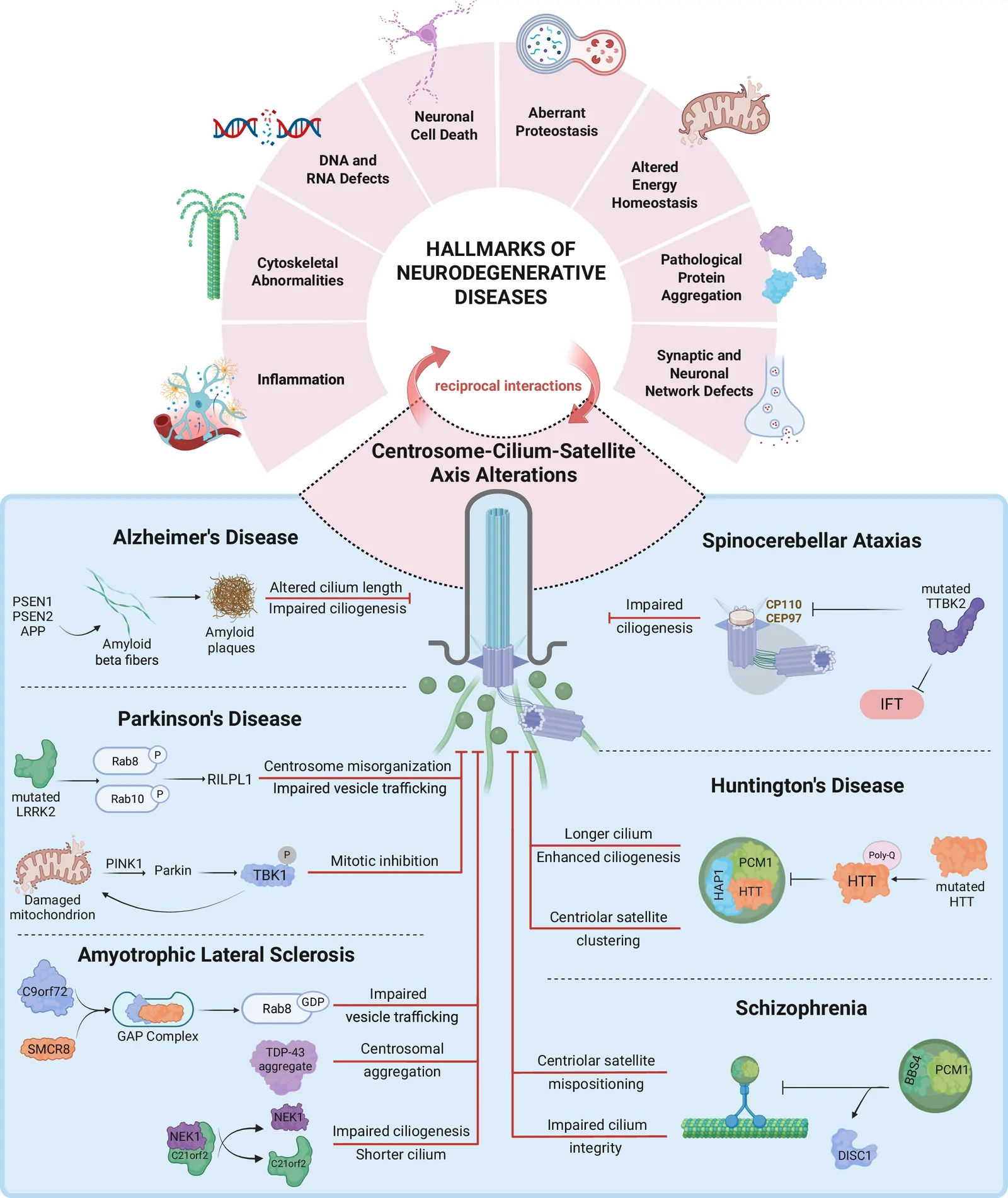
Alterations of the centrosome–cilium–satellite axis in neurodegenerative diseases (NDDs) and related neurodevelopmental contexts. The upper panel summarizes established cellular and molecular hallmarks of NDDs, including pathological protein aggregation, impaired proteostasis, altered energy metabolism, mitochondrial dysfunction, synaptic and neuronal network defects, inflammation, cytoskeletal abnormalities, and DNA/RNA defects. The centrosome–cilium–satellite axis is depicted as interacting reciprocally with these hallmarks, emphasizing that axis alterations may both respond to and influence disease-associated cellular stress pathways. The lower panel depicts selected disease-associated examples of centrosome, cilium, or satellite alterations. These examples illustrate disease-specific intersections between the axis and established pathogenic mechanisms. In Alzheimer’s disease, APP/Presenilin- and Aβ-associated models show altered ciliogenesis, ciliary length, and ciliary signaling. In Parkinson’s disease, mutant LRRK2 promotes Rab8/Rab10 phosphorylation and RILPL1-dependent defects in centrosome organization and vesicle trafficking, while PINK1/Parkin-dependent recruitment of TBK1 to damaged mitochondria links mitochondrial stress to mitotic inhibition. In amyotrophic lateral sclerosis, the C9orf72–SMCR8 GAP complex enhances Rab8 GDP formation and disrupts trafficking required for ciliogenesis; TDP-43 localizes to the pericentriolar matrix and associates with centrosome-associated RNAs and proteins; and NEK1-C21orf2 dysfunction is associated with impaired ciliogenesis and shorter motor-neuron cilia. In spinocerebellar ataxia, mutant TTBK2 prevents CP110–CEP97 removal and blocks cilium initiation and recruitment of IFT machinery to the basal body. In Huntington’s disease, mutant polyQ-expanded HTT disrupts PCM1–HAP1–HTT interactions, causing satellite clustering at basal bodies, enhanced ciliogenesis, and elongated cilia. Schizophrenia is included as a related neurodevelopmental and neuropsychiatric context, where disruption of the PCM1-DISC1-BBSome axis leads to defective centriolar satellite positioning, impaired trafficking of ciliary cargo, and compromised ciliary integrity. Created in BioRender. Begar, E. (2026) https://BioRender.com/oyxlzb1.

Alterations affecting the centrosome–cilium–satellite axis have been reported across several NDD models and selected related neurodevelopmental or neuropsychiatric contexts (Fig. 2; Table 1). These alterations arise from different disease-associated molecular changes, including pathogenic mutations or risk variants, protein misfolding or aggregation, and epigenetic dysregulation, and converge on core axis functions such as cilium assembly, centrosome organization, intracellular trafficking, ciliary signaling, satellite homeostasis, and protein quality control. Multifunctional kinases such as TTBK2, NEK1, and LRRK2 provide relatively direct links to cilium assembly or disassembly, Rab-dependent trafficking, or centrosome organization. In contrast, pleiotropic disease-linked proteins such as α-synuclein, PINK1/Parkin, Presenilins, and Huntingtin intersect with the axis more indirectly through established pathways, including proteostasis, mitochondrial quality control, vesicular trafficking, and cytoskeletal regulation. Because these processes overlap with classical NDD hallmarks, centrosome–cilium–satellite alterations may exacerbate proteostasis failure, mitochondrial stress, cytoskeletal disruption, altered signaling, and inflammation. Conversely, disease-associated stresses can further compromise ciliary structure and signaling, centrosome organization, and satellite homeostasis. Thus, this axis may both influence and be shaped by established neurodegenerative processes, rather than occupying a uniform causal position across diseases.

**Table 1:** Reported centrosome–cilium–satellite axis alterations in neurodegenerative diseases and related neuropsychiatric or neurodevelopmental contexts.

Disease	Key proteins	Localization/axis-associated link	Reported axis-associated alterations
**AD**	PSEN1/PSEN2	Centrosomes; PSEN2 at the basal body	APP/Presenilin- and Aβ-associated models show altered ciliogenesis, ciliary length, and Shh signaling
	APP/Aβ	APP at ciliary membrane; Aβ exposure/pathology in AD models	Altered neuronal and microglial ciliation; impaired ciliary signaling; ependymal motile cilia/CSF-flow defects
	Tau	Microtubules	Amyloid/tau pathology in 3xTg-AD mice is associated with shortened dentate granule neuron cilia
**PD**	LRRK2	Cytosolic/endolysosomal kinase	Phosphorylates Rab8/10/12; Rab–RILPL1/2 accumulation; impaired ciliogenesis, centrosome cohesion and MT polarity; cilia loss in striatal interneurons/astrocytes
	PINK1/Parkin	Mitochondria; TBK1 is recruited away from centrosomes during mitophagy	PINK1 loss shortens cilia and alters Shh signaling; links ciliary signaling to mitochondrial stress in PD models
	α-synuclein	Aggregates near centrosome; binds γ-tubulin and pericentrin	Overexpression/fibrils reduce ciliogenesis; disrupt MTs and polarity; induce centrosome-associated aggresomes
**ALS**	NEK1	Basal body	Abnormal/reduced cilia; impaired Shh signaling; reduced α-tubulin acetylation; Aurora A–HDAC6–dependent disassembly
	C21orf2/CFAP410	Basal body; NEK1 interactor	Reduced cilia number/length; impaired Shh signaling; defective transport; ALS variants weaken C21orf2–NEK1 interaction
	C9orf72	SMCR8–Rab8a axis	C9orf72–SMCR8 regulates Rab8a-dependent ciliogenesis; C9orf72 loss increases cilia, whereas repeat-expansion patient-derived motor neurons show reduced ciliary frequency/length
	TDP-43	Pericentriolar matrix	Localizes to centrosomes and binds centrosome-associated RNAs and proteins
	SOD1	Cytoplasm	SOD1-G93A models show reduced or lost motor-neuron cilia and altered Shh signaling
**SCAs**	TTBK2 (SCA11)	Mother centriole	Impaired CP110 removal/IFT recruitment; failed ciliogenesis; defective Shh signaling
	Ataxin-10 (SCA10)	Centrioles and midbodies	Cytokinesis defects; interacts with NPHP5/ARL13B; current evidence does not support a direct ciliogenesis defect
	Ataxin-3 (SCA3)	Basal body, photoreceptor axoneme	Regulates ciliogenesis and cilium length in photoreceptor-related contexts
	Ataxin-7 (SCA7)	Centrosomes, cilia	PolyQ-expanded Ataxin-7 is lost from photoreceptor cilia; associated with ciliary defects and photoreceptor degeneration
**HD**	HTT	Centrosomes, cilia, satellites	HTT/HAP1 loss disperses satellites and reduces ciliogenesis; polyQ-HTT causes centrosomal satellite accumulation and elongated striatal cilia
**SZ**	PCM1	Centriolar satellites	Satellite disruption; loss of neuronal cilia; ventricular enlargement; altered dopaminergic signaling
	DISC1	PCM1–BBS4-associated pathway	Cooperates with PCM1/BBS4; loss causes neuronal migration defects
	BBS4	Cilia, satellites	Neuronal migration defects; cognitive and psychiatric features reported in BBS ciliopathies
	SDCCAG8 (BBS16)	Centrosomes, transition zone	Pericentrosomal satellite clustering; neuronal polarization and migration

This table summarizes selected disease-associated proteins in Alzheimer’s disease (AD), Parkinson’s disease (PD), amyotrophic lateral sclerosis (ALS), spinocerebellar ataxias (SCAs), Huntington’s disease (HD), and schizophrenia (SZ) that localize to, interact with, or regulate centrosomes, basal bodies, primary cilia, motile cilia, or centriolar satellites. For each disease, proteins are listed together with reported localization or axis-associated link and associated cellular phenotypes. The entries highlight reported intersections between disease-associated pathways and the centrosome–cilium–satellite axis, but should not be interpreted as evidence for equivalent causal strength across diseases. In many contexts, the evidence remains model-dependent, correlative, or limited to specific genetic backgrounds, cell types, or disease stages.

The strength and functional consequences of these links vary substantially across diseases, cell types, disease stages, and individual components of the axis. Centrosomal and ciliary phenotypes are currently more broadly documented across classical NDD models, whereas satellite-specific mechanisms remain less extensively characterized and are clearest in selected contexts, particularly Huntington’s disease and schizophrenia-related neurodevelopmental models. We therefore evaluate the disease literature by considering evidence type and biological context, including direct genetic links, patient-derived cellular models, animal models, and observations from human tissue. This framework helps distinguish cases in which centrosome–cilium–satellite alterations may act as early contributors from those in which they modify established pathology, reflect adaptive or compensatory responses to cellular stress, or amplify late-stage degeneration.

In the following sections, we examine reported axis alterations in AD, PD, ALS, hereditary ataxias, and Huntington’s disease. We discuss schizophrenia separately as a related neurodevelopmental and neuropsychiatric context because it provides particularly informative evidence for satellite-associated regulation of ciliary integrity, neuronal organization, ventricular morphology, and dopaminergic signaling

### AD

AD is the most prevalent NDD and the leading cause of dementia worldwide (Knopman et al, 2021). Clinically, it manifests as progressive memory loss, cognitive decline, and behavioral changes. Most AD cases are late-onset and sporadic, whereas rare early-onset familial forms are mainly caused by autosomal-dominant mutations in genes encoding amyloid precursor protein (APP) and presenilins 1 and 2 (PSEN1 and PSEN2) (Knopman et al, 2021). The pathological hallmarks of AD are extracellular amyloid-β (Aβ) plaques, formed from Aβ peptides generated by sequential cleavage of APP, and intracellular neurofibrillary tangles composed of hyperphosphorylated tau, a microtubule-associated protein essential for axonal stability (Kamatham et al, 2024). These aggregates impair neuronal proteostasis and synaptic function, ultimately leading to synaptic loss and neurodegeneration (Kamatham et al, 2024). In parallel, they activate glial cells and induce chronic neuroinflammatory responses that further amplify neuronal stress and injury.

Ciliary defects have emerged as a feature of AD-related cellular and animal models, particularly in contexts involving APP processing, Aβ exposure, and amyloid pathology (Fig. 2). PSEN1 and PSEN2, which are mutated in most autosomal-dominant early-onset familial AD cases, form the catalytic core of the γ-secretase complex that sequentially cleaves APP to Aβ (De Strooper et al, 1998). Disease mutations shift this cleavage toward the aggregation-prone Aβ₄₂ isoform (Chang et al, 2015). Both presenilins localize to centrosomes, and PSEN2 is enriched at the basal body of the primary cilium, placing APP-processing machinery near the ciliary base (Li et al, 1997; Ezratty et al, 2016). APP also localizes to the ciliary membrane with the Shh pathway component Smoothened (Smo), and Aβ exposure impairs cilium formation, ciliary length, and Shh signaling (Vorobyeva and Saunders, 2018). These findings provide a mechanistic link between APP/Presenilin-dependent Aβ biology and ciliary structure or signaling in AD-related cellular systems.

In vivo AD models extend this link, but also show that ciliary phenotypes are not uniform and vary with genotype, brain region, plaque proximity, and disease stage (Huang et al, 2025a; Ma et al, 2022). In 3xTg-AD mice, which develop both Aβ and tau pathology, primary cilia on dentate granule neurons are shorter (Chakravarthy et al, 2012; Ma et al, 2022). In contrast, models that exhibit Aβ pathology without tau aggregation, such as APP/PS1, 5×FAD, and the AppNL-G-F knock-in line, show region-specific changes. Neuronal cilia near plaques are shortened and fail to elongate with age in the hippocampus, whereas cilia in plaque-distant regions are elongated (Guo et al, 2025; Hu et al, 2017; Kobayashi et al, 2022). These structural changes are accompanied by altered ciliary signaling. For example, expression of ciliary GPCRs such as SSTR3 is increased in several AD models, and pharmacological modulation of the 5-HT6 receptor, another ciliary GPCR, has been shown to improve cognition in APP/PS1 mice (Guo et al, 2025; Hu et al, 2017). Together, these studies indicate that neuronal cilia are sensitive to AD-associated pathology, while emphasizing that ciliary length and signaling changes are context-dependent rather than a single uniform AD phenotype.

Beyond neurons, ciliary alterations in AD extend to glial and ependymal cells. In Aβ-driven models, microglial cilia show structural and functional remodeling. 5×FAD mice exhibit fewer and shorter cilia in the lateral septum and substantial loss of ciliated microglia in cortical regions, changes that correlate with local Aβ burden and tissue environment (Yeo et al, 2023). Loss of cilia alters microglial secretory profiles in wild-type microglia, while in 5×FAD mice, reduced ciliation is associated with greater amyloid plaque expansion, linking ciliary integrity to extracellular proteostasis across models (Yeo et al, 2023). Inflammatory signaling also feeds back onto ciliary structure, as activation of pathways such as TLR4 shortens cilia and increases pro-inflammatory activity (Baek et al, 2017). These findings link microglial cilia to inflammatory regulation and extracellular proteostasis in Aβ-driven models.

Ependymal cells lining the ventricles provide an additional AD-relevant context because their motile cilia drive cerebrospinal fluid (CSF) flow. Aβ₁-₄₂ exposure impairs ependymal ciliary beating and reduces CSF flow in ventricular explants (Makibatake et al, 2023). Because ependymal cilia generate directional flow that supports ventricular homeostasis and contributes to CSF-interstitial fluid exchange and waste clearance, Aβ-induced impairment of ciliary beating may disrupt local CSF dynamics and clearance in periventricular regions, thereby increasing tissue stress (Nelles and Hazrati, 2022). This motile cilia pathway is relevant to AD-associated clearance biology but is distinct from primary-cilium defects described in neurons and glia.

Overall, AD models reveal centrosome–cilium involvement at several levels, including APP/Presenilin-associated pathways, neuronal ciliary structure and signaling, microglial inflammatory responses, and ependymal CSF flow. However, the causal position of these changes remains unresolved. In familial APP/PSEN-based and Aβ-driven systems, ciliary defects may contribute to altered signaling, extracellular proteostasis, and inflammatory responses. In sporadic AD, they may also arise downstream of amyloid, tau, aging-related stress, or neuroinflammation. Importantly, current evidence for centrosome–cilium dysfunction in AD is strongest in cellular and animal models, whereas large-scale sporadic AD genetic studies have not yet established core ciliogenesis pathways as a major risk category (Bellenguez et al, 2022; Kunkle et al, 2019). Thus, centrosome–cilium dysfunction in AD is best viewed as an emerging, context-dependent phenotype that intersects with established AD mechanisms rather than as a primary driver of disease.

### PD

PD is a progressive NDD primarily characterized by motor dysfunction, but it also involves non-motor symptoms such as cognitive decline (Aarsland et al, 2021) (Poewe et al, 2017). It is the second most common NDD after AD. The pathological hallmark of PD is the progressive loss of dopaminergic neurons in the substantia nigra, a midbrain region essential for motor control, and the accumulation of Lewy bodies (LBs), cytoplasmic inclusions composed mainly of misfolded α-synuclein (Poewe et al, 2017). Most PD cases are sporadic, whereas an estimated 5–10% are monogenic and include genes linked to α-synuclein proteostasis, mitochondrial quality control, vesicular and endolysosomal trafficking, and autophagy-lysosomal function (Coukos and Krainc, 2024). Monogenic PD includes autosomal-dominant forms caused by mutations or multiplications of *SNCA*, which encodes α-synuclein, and pathogenic variants in *LRRK2*, which encodes leucine-rich repeat kinase 2 (Jia et al, 2022). LRRK2 is one of the most common monogenic causes of PD and the most common autosomal-dominant cause of familial PD (Ge et al, 2020; Poewe et al, 2017; Stocchi et al, 2024). Autosomal-recessive early-onset forms are associated with mutations in PRKN, which encodes the E3 ubiquitin ligase Parkin, and PINK1, which encodes a mitochondrial kinase that recruits Parkin to damaged mitochondria (Deas et al, 2011; Kitada et al, 1998).

Ciliary and centrosomal alterations have emerged as recurrent features in PD models and human tissue, particularly in genetically defined contexts (Fig. 2). Primary cilia coordinate developmental pathways important for dopaminergic neuron specification and maintenance, including Shh, FGF8, and Wnt signaling (Britto et al, 2002; Arenas, 2014). In the mature brain, many striatal GABAergic neurons have cilia enriched with dopamine receptors, especially the D1 subtype, which mediates dopaminergic signaling (Stubbs et al, 2022). In the mature brain, striatal GABAergic neurons contain cilia enriched in dopamine receptors, and dopaminergic depletion can elongate these cilia, whereas receptor activation shortens them (Miyoshi et al, 2014; Mustafa et al, 2021). Experimental and pathological evidence further support a functional link. 6-hydroxydopamine lesions elongate striatal neuronal cilia, loss of primary cilia in MPTP-treated dopaminergic neurons worsens degeneration and motor deficits, and Shh/Gli activation is protective in rodent PD models (Miyoshi et al, 2014; Bae et al, 2019; Shao et al, 2017). Consistent with these findings, postmortem analyses of human PD brains reveal reduced cilia number and structural defects of cilia (Tian et al, 2024). Together, these findings indicate that ciliary structure and signaling are sensitive to dopaminergic state and may influence dopaminergic neuron maintenance in specific PD models.

Among PD-linked mechanisms, LRRK2 provides one of the clearest connections to centrosome–cilium regulation (Alessi and Sammler, 2018). Pathogenic *LRRK2* mutations such as R1441C and G2019S hyperactivate LRRK2 kinase activity, particularly toward RAB8A, RAB10, and RAB12, which regulate ciliary membrane trafficking and centrosome organization (Aarsland et al, 2021; Li et al, 2024; Steger et al, 2017; Steger et al, 2016). Hyperphosphorylated RABs recruit effectors such as RILPL1, RILPL2, JIP3 and 4, and Myosin Va, driving their abnormal accumulation at centrosomes and leading to defects in ciliogenesis, vesicle transport, and centrosome cohesion. In vivo, LRRK2 hyperactivation causes loss of primary cilia in striatal cholinergic interneurons and astrocytes, accompanied by impaired Shh signaling and altered expression of *Gli1* and *Gdnf*, both critical for dopaminergic neuron maintenance (Dhekne et al, 2018; Khan et al, 2021). LRRK2-driven centrosomal abnormalities also disrupt microtubule orientation and polarity, compromising neurite outgrowth and axonal architecture. Importantly, restoring RAB8a-dependent trafficking through LRRK2 kinase inhibition rescues ciliary and centrosomal defects in cell models, supporting LRRK2-dependent PD models as a tractable setting for testing whether ciliary rescue modifies disease-relevant phenotypes (Dhekne et al, 2018; Dhekne et al, 2021; Fdez et al, 2022).

Another PD-linked kinase, PINK1, connects mitochondrial homeostasis with centrosome and cilium biology. Under normal conditions, PINK1 localizes to mitochondria, where it mediates mitophagy through recruitment of the E3 ubiquitin ligase PARKIN (Deas et al, 2011; Vargas et al, 2023). Upon mitochondrial damage, PINK1 accumulates on the outer membrane and activates PARKIN, which promotes TBK1 recruitment to damaged mitochondria (Sarraf et al, 2019). This relocalization depletes TBK1 from centrosomes and suppresses mitotic signaling, linking mitochondrial quality control to centrosome- and cell cycle-regulation. Beyond centrosomes, PINK1 is also implicated in cilium formation and signaling. In PINK1-deficient human cellular and mouse models, primary cilia are shortened, and inhibition of excessive Shh signaling restores both cilium length and mitochondrial function, indicating that dysregulated ciliary signaling can contribute to mitochondrial stress in PD models (Schmidt et al, 2022). In *Pink1* knockout mice, striatal cholinergic interneurons and astrocytes similarly display shortened cilia and impaired Shh-dependent induction of Gdnf transcription (Bagnoli et al, 2025). Finally, *PINK1* and *LRRK2* mutations do not produce additive phenotypes, suggesting that these kinases may converge on overlapping cilium-associated neuroprotective pathways in the brain (Bagnoli et al, 2025).

Beyond LRRK2 and PINK1, α-synuclein pathology further implicates the centrosome and cilium dysfunction in PD. Overexpression of α-synuclein or its familial PD-associated mutants in differentiated in vitro neuroblastoma cell models induces aggresome formation, accompanied by decreased ciliogenesis, disrupted microtubule organization, and loss of neuronal polarity (Iqbal et al, 2020). Similarly, in vivo, α-synuclein overexpression in zebrafish embryos reduces the number and length of olfactory cilia in dopaminergic neurons (Iqbal et al, 2020). In primary cortical neurons treated with α-synuclein preformed fibrils, activation of the Shh pathway enhances ciliogenesis and suppresses α-synuclein aggregation, indicating that ciliary signaling can mitigate proteostatic stress (Quansah et al, 2025). Supporting this, loss of the DNA methylation regulator TET2 promotes ciliogenesis and upregulation of Shh signaling genes, which is associated with decreased α-synuclein accumulation and protection of dopaminergic neurons in a mouse synucleinopathy model (Luk et al, 2012; Marshall et al, 2020; Quansah et al, 2025). These findings suggest a reciprocal relationship between α-synuclein proteostasis and ciliary signaling, while leaving open how much of α-synuclein-dependent PD pathology is mediated through ciliary mechanisms versus broader proteostatic and trafficking pathways.

Beyond its effects on cilia, α-synuclein also interferes with centrosome-based proteostasis. Lewy bodies (LBs), intraneuronal α-synuclein inclusions, often form near the centrosome and sequester centrosomal proteins such as γ-tubulin and Pericentrin (McNaught et al, 2002). Their composition and localization resemble aggresomes, cytoplasmic structures that assemble at the centrosome to sequester misfolded or ubiquitinated proteins for degradation via autophagy (Johnston et al, 1998). Consistently, proteasome inhibition or overproduction of misfolded proteins in dopaminergic neurons induces aggresome or LB-like inclusions with associated cytotoxicity (McNaught et al, 2002). Under proteostatic stress, Parkin and poly-ubiquitinated substrates accumulate at aggresomes in a γ-tubulin-dependent manner, suggesting that the centrosome acts as a platform where Parkin mediates ubiquitination and clearance of protein aggregates implicated in PD (Zhao et al, 2003). When centrosome-associated quality control pathways are overwhelmed by excess α-synuclein or impaired Parkin function, proteotoxic stress may increase and contribute to neuronal vulnerability.

In summary, PD-associated mechanisms involving LRRK2 hyperactivation, PINK1/Parkin dysfunction, and α-synuclein aggregation intersect with centrosome and cilium biology through Rab-dependent trafficking, mitochondrial quality control, ciliary signaling, microtubule organization, and centrosome-associated proteostasis. The strongest mechanistic evidence comes from LRRK2- and PINK1-dependent models in which ciliary phenotypes are linked to signaling and mitochondrial readouts, whereas α-synuclein studies highlight reciprocal interactions between ciliary signaling and proteostatic stress. Ciliary phenotypes in PD models are not uniform, with studies reporting reduced ciliogenesis, altered ciliary length, or changes in signaling responsiveness depending on genetic background, neuronal subtype, model system, and disease stage. Future work should determine whether restoring centrosome or cilium function produces disease-relevant benefit independently of, or in combination with, broader effects on mitochondrial homeostasis, lysosomal trafficking, and α-synuclein proteostasis.

### ALS

ALS is the most common adult-onset motor neuron disease, characterized by progressive degeneration of upper and lower motor neurons. It is a severe, rapidly progressing neuromuscular condition that typically leads to death within 3-4 years of symptom onset, primarily due to retrograde degeneration of brainstem and spinal α-motoneurons (αMNs) (Mead et al, 2023; Nguyen et al, 2018; Petrov et al, 2017; Taylor et al, 2016). Approximately 5–10% of ALS cases are familial, following Mendelian inheritance, while the remaining 90–95% are sporadic (Hardiman et al, 2011). The genetic landscape of ALS is highly complex, including more than 30 implicated genes, including *SOD1*, *C9orf72*, *FUS*, *TARDBP*, and *NEK1*, which encode structurally and functionally diverse proteins involved in RNA metabolism, protein homeostasis, cytoskeletal regulation, and organelle maintenance (Gregory et al, 2020; Nguyen et al, 2018). This diversity underscores the mechanistic heterogeneity of ALS, and despite decades of research, therapeutic options remain limited and largely ineffective (Mead et al, 2023; Nguyen et al, 2018; Petrov et al, 2017).

Although protein aggregation and misfolded protein stress are pathological hallmarks of ALS, affected motor neurons also exhibit RNA processing defects, impaired proteostasis, cytoskeletal disruption, mitochondrial stress, and genomic instability (Malik and Wiedau, 2020). This mechanistic breadth makes ALS a particularly informative setting for examining how centrosome–cilium–satellite biology intersects with motor neuron vulnerability. Several ALS-linked genes, including *NEK1*, *C21orf2*, *C9orf72*, and *SOD1*, have now been connected to ciliary structure, ciliary signaling, trafficking, or centrosome-associated functions, whereas TDP-43 pathology provides a complementary link between RNA dysregulation and centrosome-localized transcripts or proteins (Takahashi et al, 2025). The nature of these links differs across ALS contexts. NEK1 and C21orf2 provide relatively direct genetic and model-based connections to centrosome–cilium regulation, whereas C9orf72, TDP-43, and SOD1 connect this axis to broader disturbances in trafficking, RNA metabolism, proteostasis, oxidative stress, or cytoskeletal regulation. In the following sections, we examine genetically and pathologically distinct ALS contexts to define where centrosome–cilium defects may act as early contributors, disease modifiers, or downstream consequences of broader motor neuron stress (Fig. 2).

#### NEK1-linked ALS

One of the most compelling examples of centrosomal and ciliary dysfunction in ALS involves mutations in *NEK1* (NIMA-related kinase 1). First identified as a genetic risk factor in 2016, *NEK1* mutations now account for ~3–5% of all ALS cases(Brenner et al, 2016; Kenna et al, 2016; Rifai et al, 2025; Yao et al, 2021). Most variants are monoallelic loss-of-function mutations, although emerging evidence suggests that toxic gain-of-function mechanisms may also contribute to disease progression (Brenner et al, 2016; Georgiadou et al, 2026; Mann et al, 2023; Rifai et al, 2025). *NEK1* encodes a multifunctional serine/threonine kinase that regulates DNA damage response, cell cycle regulation, mitochondrial activity, and cytoskeletal organization (Moniz et al, 2011). Recent studies using patient-derived and engineered ALS models implicate ciliary defects as a factor in NEK1-linked ALS pathogenesis (Georgiadou et al, 2026; Gregorczyk et al, 2023; Noh et al, 2025; Watanabe et al, 2025).

NEK1’s role in ciliogenesis predates its association with ALS. Nek1-deficient kat2J mice develop structurally abnormal primary cilia and polycystic kidney disease (Chen et al, 2014; Upadhya et al, 2000). *NEK1* mutations were later identified in human ciliopathies such as short-rib thoracic dysplasia and oral-facial-digital syndrome type II (El Hokayem et al, 2012; Monroe et al, 2016; Thiel et al, 2011). These findings establish NEK1 as a ciliary protein whose mutation spectrum spans ciliopathies and ALS.

Mechanistically, NEK1 localizes to the basal body and regulates cilium assembly and stability. While overexpression of NEK1 in epithelial cells inhibits cilium formation, its depletion in fibroblasts reduces the number of ciliated cells, indicating that balanced NEK1 activity is required for proper ciliogenesis (Chen et al, 2014; Shalom et al, 2008; White and Quarmby, 2008). Although the precise mechanisms by which NEK1 exerts this function remain incompletely understood, domain-mapping analyses show that its kinase activity and coiled-coil domain containing a ciliary-targeting sequence are essential for proper ciliogenesis (White and Quarmby, 2008). Moreover, mass spectrometry and biochemical analyses show that NEK1 interacts with ciliary transport components such as KIF3A and phosphorylates α-tubulin and Importin-β1, linking its kinase activity to microtubule stabilization and nucleocytoplasmic transport (Mann et al, 2023). Together, these findings establish NEK1 as a regulator of cilium structure and function.

Recent patient-derived and experimental model studies have revealed the cellular consequences of *NEK1* mutations in ALS. Fibroblasts from ALS patients carrying *NEK1* loss-of-function mutations and iPSC-derived motor neurons depleted of NEK1 show abnormal primary cilia, impaired Shh signaling, and reduced tubulin acetylation (Noh et al, 2025). These defects are accompanied by elevated intracellular calcium that activates the Aurora A-HDAC6-dependent cilium disassembly and aberrant cell-cycle re-entry. Importantly, treatment with a calcium chelator or HDAC6 inhibitor restores these ciliary defects, supporting NEK1-linked ALS as a strong preclinical context for testing whether ciliary stabilization modifies motor neuron phenotypes. A complementary study using multiple iPSC-derived motor neuron models, including *NEK1* knockdown, kinase inhibition, and patient-derived mutations, identified broader disruptions in microtubule homeostasis and nucleocytoplasmic transport (Mann et al, 2023).

Consistent with these defects, in vivo models demonstrate that loss or truncation of NEK1 impairs both neuronal function and cilium-dependent processes. Silencing of Niki, the NEK1 homolog in Drosophila, leads to progressive motor dysfunction and reduced survival, while mice expressing a truncated form of Nek1 develop motor deficits together with ciliopathy-associated phenotypes such as polycystic kidneys, skeletal abnormalities, and male infertility (Georgiadou et al, 2026; Mann et al, 2023). Notably, this truncated Nek1 variant exhibits both gain- and loss-of-function characteristics, forming nuclear aggregates and becoming delocalized from its physiological sites (Georgiadou et al, 2026). Collectively, these studies support NEK1 dysfunction as a cause of ciliary architecture, microtubule organization, and motor neuron homeostasis defects in ALS models. Because NEK1 also regulates DNA damage response, mitochondrial activity, cytoskeletal organization, and nucleocytoplasmic transport, future work should determine how much of NEK1-linked motor neuron vulnerability is driven directly by ciliary dysfunction versus broader NEK1-dependent cellular pathways.

#### C21orf2-linked ALS

C21orf2 (also known as CFAP410) has emerging roles in ciliogenesis, mitochondrial homeostasis, and genome maintenance (Gregorczyk et al, 2023). It was first identified in functional siRNA screens for regulators of cilium formation, and its mutations have been implicated in a spectrum of ciliopathies, including retinal dystrophy with variable skeletal involvement (Abu-Safieh et al, 2013; Lai et al, 2011; Wheway et al, 2015). Comparative interactome analyses of five ALS-associated proteins identified C21orf2 as a highly connected factor with multiple neuronal binding partners, suggesting a central role in pathways relevant to motor neuron maintenance (Zelina et al, 2024). Structural and biochemical studies have shown that C21orf2 and NEK1 form a physical complex with functions in ciliary regulation and neuronal survival (Gregorczyk et al, 2023; Wheway et al, 2015).

At the cellular level, C21orf2 localizes to the basal body and cooperates with NEK1 to regulate primary cilium formation and signaling. Loss of C21orf2, as well as ALS-associated C21orf2 variants that weaken its interaction with NEK1, reduce the number and length of primary cilia. iPSC-derived motor neurons from ALS patients carrying *C21orf2* mutations exhibit similar ciliary defects, recapitulating *NEK1* loss-of-function phenotypes (De Decker et al, 2025; Gregorczyk et al, 2023). This ciliary dysfunction leads to impaired Shh signaling, defective ciliary transport, and downregulation of cilia-dependent targets such as CRABP1, a protein required for motor neuron survival (De Decker et al, 2025). Notably, these ciliary defects are accompanied by a reduced capacity for neuromuscular junction formation. In vivo, knockdown of *C21orf2* orthologs in Drosophila and zebrafish leads to locomotor defects, increased neuronal apoptosis, and male infertility, phenotypes consistent with both ciliopathies and ALS (De Decker et al, 2025; Zelina et al, 2024).

Beyond ciliogenesis, C21orf2 also functions in DNA damage response. Its loss leads to accumulation of DNA breaks and defective homologous recombination, defects that can be rescued by *NEK1* overexpression, further supporting that both proteins act within a shared repair pathway (Fang et al, 2015; Zelina et al, 2024). In the context of ALS, the C21orf2-V58L variant further disrupts this pathway, altering DNA damage signaling and reducing NEK1 expression in motor neurons (Zelina et al, 2024). Together, these findings support C21orf2 as a multifunctional regulator of ciliary signaling and genome maintenance that cooperates with NEK1, making the NEK1-C21orf2 module a strong example of the link between centrosome–cilium dysfunction and ALS-associated motor neuron vulnerability.

#### C9orf72-linked ALS

Chromosome 9 open reading frame 72 (*C9orf72*) is the most frequently mutated gene in ALS and a major contributor to both familial and sporadic cases. Its pathogenic GGGGCC hexanucleotide repeat expansion in the first intron accounts for about 50% of familial and 5–10% of sporadic ALS cases (Majounie et al, 2012; Renton et al, 2011). This expansion causes a toxic gain of function through sequestration of RNA-binding proteins and the accumulation of toxic dipeptide repeat proteins, together with loss of C9orf72 function (Aladesuyi Arogundade et al, 2021; Haeusler et al, 2014; Renton et al, 2011; Shi et al, 2018). Functionally, C9orf72 regulates autophagy, lysosomal activity, immune signaling, synaptic transmission, and membrane trafficking (Haeusler et al, 2014; Nijs and Van Damme, 2024; Renton et al, 2011). The protein’s DENN (Differentially Expressed in Normal and Neoplastic cells) domain identifies it as a guanine nucleotide exchange factor (GEF) for Rab GTPases that regulate both vesicle transport and autophagy, positioning C9orf72 at the interface of these processes.

Recent evidence indicates that C9orf72 regulates ciliogenesis through its interaction with SMCR8. Together, C9orf72 and SMCR8 form a GTPase-activating protein (GAP) complex that acts on RAB8A, a small GTPase essential for vesicle trafficking during ciliogenesis (Tang et al, 2023). By promoting RAB8A-GTP hydrolysis, C9orf72–SMCR8 GAP complex functions as a negative regulator of ciliogenesis and Shh signaling, thereby limiting cilium formation and length under physiological conditions. Consistently, *C9orf72* knockout mice show increased ciliary number and length across multiple tissues, including neurons and astrocytes, showing that loss of C9orf72 enhances ciliogenesis in vivo (Tang et al, 2023). In contrast, motor neurons derived from ALS patient iPSCs carrying C9orf72 repeat expansions exhibit reduced ciliary frequency and length (De Decker et al, 2025). This difference may reflect the combined effects of C9orf72 loss-of-function and toxic gain-of-function mechanisms, which can affect vesicle trafficking, autophagy, and ciliogenesis in different ways depending on context.

Genetic studies further suggest interactions between *C9orf72* and other ALS-associated genes. For example, mutations in *C9orf72* can co-occur with variants in *NEK1*, indicating partial convergence of their cellular pathways (Nguyen et al, 2018; Riva et al, 2022). In patient-derived iPSC motor neurons harboring both *C9orf72* repeat expansions and *NEK1* haploinsufficiency, DNA damage is exacerbated, whereas ciliary defects are not further worsened (Santangelo et al, 2024). This suggests that NEK1 may modify genomic instability while the ciliary phenotypes associated with C9orf72 dysfunction are regulated independently. Together, these findings identify C9orf72 as a regulator of Rab-dependent trafficking and ciliogenesis whose ALS-associated disruption can alter ciliary signaling, genome maintenance, and neuronal homeostasis. The divergent ciliary phenotypes observed in knockout and patient-derived models further emphasize that C9orf72-related ciliary defects depend on the balance between loss-of-function and repeat-expansion-associated toxic mechanisms.

#### TDP-43 pathology and ALS

TAR DNA-binding protein 43 (TDP-43) pathology is a defining molecular hallmark of ALS, detected in approximately 97% of cases across diverse genetic backgrounds (Arai et al, 2006; Ling et al, 2013; Mackenzie et al, 2007; Neumann et al, 2006; Sreedharan et al, 2008). Cytoplasmic translocation and aggregation of phosphorylated and ubiquitinated TDP-43 in degenerating neurons is a pathological hallmark of TDP-associated diseases (Jo et al, 2020; Prasad et al, 2019; Suk and Rousseaux, 2020). TDP-43, encoded by the *TARDBP* gene, is a highly conserved and ubiquitously expressed RNA-binding protein that normally resides in the nucleus, where it regulates transcription, splicing, mRNA transport, and stability. Mutations in *TARDBP* cause familial forms of ALS (Kabashi et al, 2008; Sreedharan et al, 2008). Notably, TDP-43 pathology often coexists with mutations in other ALS-associated genes such as *NEK1*, *C9orf72*, and *C21orf2*, whose functions intersect with the centrosome–cilium–satellite axis, highlighting convergence between RNA dysregulation and organelle dysfunction in ALS (Cook et al, 2020; Lee et al, 2019; Rifai et al, 2024; Riva et al, 2022; Watanabe et al, 2025).

Recent studies show that TDP-43 localizes to the pericentriolar matrix of the centrosome across the cell cycle, expanding its distribution beyond the nucleus and cytoplasm (Bodin et al, 2023). This localization is notable because the centrosome contains ribosomes, RNA-binding proteins, and specific transcripts that support localized translation, several of which have been identified as TDP-43 interactors (Chouaib et al, 2020; Safieddine et al, 2021; Sepulveda et al, 2018). Several centrosomal mRNAs, including *PCNT*, *NUMA1*, *ASPM*, and *CEP350*, contain TDP-43 binding sites, and proteomic analyses identified centrosomal TDP-43 interactors, including ALS-linked proteins such as Ataxin-2, CCNF, DCTN1, PFN1, and VCP (Bodin et al, 2023). Although the precise role of centrosomal TDP-43 remains undefined, these findings suggest that TDP-43 may regulate the localization, stability, or translation of centrosome-associated transcripts or proteins. Because several of these interactors regulate microtubule organization, centrosome structure, or cilium assembly, centrosomal TDP-43 may connect RNA dysregulation to centrosome–cilium-associated defects in ALS. However, whether TDP-43 pathology directly disrupts centrosome–cilium function, or whether centrosomal changes arise secondarily from broader RNA-processing, proteostatic, or cytoskeletal stress, remains to be tested in disease-relevant neuronal models.

#### SOD1-linked ALS

*SOD1*-mutant ALS represents a distinct proteinopathy defined by SOD1-positive inclusions, in contrast to most ALS cases characterized by TDP-43 pathology (Mackenzie et al, 2007). *SOD1*, encodes a copper/zinc superoxide dismutase critical for redox homeostasis and was the first ALS gene identified in 1993 (Rosen et al, 1993). More than 200 pathogenic variants have since been reported, accounting for ~10–20% of familial ALS and 1.5% of sporadic cases globally (Benatar et al, 2025; Gregory et al, 2020; Malik and Wiedau, 2020). Across these mutations, misfolding and aggregation of SOD1 drive a toxic gain-of-function associated with oxidative stress, impaired axonal transport, neuroinflammation, and mitochondrial dysfunction (Mead et al, 2023). Therapeutically, the antisense oligonucleotide tofersen, which promotes degradation of mutant *SOD1* mRNA, reduces SOD1 protein levels and improves disease biomarkers in preclinical models and human clinical trials (Everett and Bucelli, 2024; McGuigan and Blair, 2025).

*SOD1*-driven pathology is multifactorial and also intersects with primary cilium biology. In the widely used *SOD1*-G93A mouse model, primary cilia on spinal motor neurons are reduced or lost in adult mice, implicating ciliary dysfunction in SOD1-associated ALS (Ma et al, 2011). In cellular models expressing mutant SOD1, Shh pathway activation protects against oxidative stress, whereas pathway inhibition increases susceptibility to injury (Peterson and Turnbull, 2012). Consistently, in *SOD1*-G93A mice, pharmacological activation of Shh signaling prolongs survival, whereas Shh inhibition shortens survival; these effects are associated with PI3K/AKT signaling (Qi et al, 2022). Together, these findings suggest that motor-neuron ciliation and Shh signaling can influence disease severity in SOD1-linked ALS models. However, it remains unclear whether SOD1 mutations drive disease through primary cilium defects, or whether reduced ciliation and altered Shh signaling instead act as propagating factors or downstream consequences of oxidative stress, mitochondrial dysfunction, axonal pathology, and proteostatic failure.

### Spinocerebellar ataxias

Spinocerebellar ataxias (SCAs) are a genetically heterogeneous group of inherited, usually autosomal-dominant, progressive NDDs characterized by cerebellar dysfunction, leading to impaired coordination, gait instability, and dysarthria (slurred or slow speech) (Cui et al, 2024). The first form to be genetically identified was SCA1, and since then more than 40 subtypes have been described, with SCA2, SCA3, and SCA7 among the most common in Europe (Coarelli et al, 2023; Klockgether et al, 2019; Sullivan et al, 2019). Many SCAs arise from mutations in ATXN genes encoding ataxin proteins, which regulate RNA metabolism, protein homeostasis, transcription, and cytoskeletal organization (Coarelli et al, 2023; Paulson, 2009; Tezenas du Montcel et al, 2014). For several frequent subtypes, CAG trinucleotide repeat expansions generate polyglutamine (polyQ)-expanded proteins prone to misfolding, aggregation, and toxic gain-of-function effects (Paulson, 2009). SCA pathology also involves transcriptional dysregulation, defective autophagy, ion-channel dysfunction, and mitochondrial impairment (Matilla-Duenas et al, 2010).

Several SCA-associated proteins intersect with centrosomal or ciliary pathways, but the strength of evidence differs substantially across subtypes. A well-established link between SCAs and ciliogenesis is SCA11, which is caused by mutations in TTBK2 (Tau tubulin kinase 2), a critical regulator of ciliogenesis. TTBK2 is recruited to the distal appendages of the mother centriole, where it removes CP110 and recruits IFT components to initiate ciliogenesis (Bowie and Goetz, 2020; Bowie et al, 2018; Goetz et al, 2012). Disease-associated mutations impair cilium assembly and disrupt ciliary signaling. Mouse models engineered to carry SCA11-associated Ttbk2 mutations fail to form primary cilia, display disrupted Shh signaling, and develop Purkinje-cell degeneration and motor ataxia (Bowie et al, 2018). Conditional deletion of *Ttbk2* or the ciliogenesis gene *Ift88* in the adult cerebellum produces similar neurodegeneration, supporting a causal and potentially initiating role for impaired cilium-dependent signaling in Purkinje-cell vulnerability in this model (Bowie and Goetz, 2020).

Other ataxin proteins show more context-specific links to centrosomal, ciliary, or cytoskeletal regulation. Ataxin-10, the causal protein in SCA10, localizes to centrioles and midbodies. It interacts with the mitotic kinase Aurora B, which phosphorylates Ataxin-10 to promote binding to Polo-like kinase 1 (PLK1), a key regulator of cytokinesis (Tian et al, 2015). Loss of Ataxin-10 disrupts cytokinesis and induces apoptosis in dividing and cerebellar neurons (Marz et al, 2004). Ataxin-10 also interacts with ciliopathy-associated proteins, including the transition-zone protein NPHP5 and the ciliary GTPase ARL13B, which are both critical for ciliogenesis (Bentley-Ford et al, 2021; Cevik et al, 2013; Sang et al, 2011). Although *ATXN10* mutations are linked to ciliopathy syndromes such as nephronophthisis and Joubert syndrome, its loss does not impair cilium formation or morphology in vitro or in vivo (Bentley-Ford et al, 2021). Thus, Ataxin-10 may contribute to centrosomal or ciliary regulation through protein interactions and cytokinesis-related functions, but current evidence does not support a direct ciliogenesis defect comparable to TTBK2.

Additional ataxins further illustrate subtype-specific connections to cilia and cytoskeletal organization. The protein product of *ATXN3*, the causal gene in SCA3, localizes to the basal body and axoneme of photoreceptor cilia and regulates ciliogenesis and maintains proper cilium length (Toulis et al, 2020). Ataxin-7, mutated in SCA7, localizes to centrosomes and multiple types of cilia, including photoreceptor cilia. In zebrafish, Atxn7 loss causes ciliary defects and photoreceptor degeneration, and in SCA7 mouse models, polyQ-expanded Ataxin-7 is progressively lost from photoreceptor cilia, correlating with retinal pathology (Orr, 2012). Cytoplasmic Ataxin-7 also associates with microtubules and stabilizes them against depolymerization, and ATXN2 orthologs in *C. elegans* regulate centrosome size and microtubule nucleation, indicating a broader role in cytoskeletal regulation (Nakamura et al, 2012; Stubenvoll et al, 2016).

Together, these studies indicate that centrosomal and ciliary pathways are relevant to selected SCA subtypes, but the strength and nature of the evidence vary. TTBK2/SCA11 provides the clearest direct link to impaired ciliogenesis and Purkinje-cell vulnerability, whereas other ataxins connect to the axis through more specialized effects on photoreceptor cilia, cytokinesis, or microtubule organization. This subtype-specific pattern is consistent with the cerebellum’s dependence on ciliary signaling during development and neuronal maintenance, but cerebellar degeneration in SCAs should also be interpreted alongside established mechanisms such as polyQ toxicity, transcriptional dysregulation, mitochondrial impairment, defective proteostasis, and ion-channel dysfunction (Kumar et al, 2023).

### Huntington’s disease

Huntington’s disease (HD) is a progressive and fatal NDD characterized by motor dysfunction and cognitive decline. Like spinocerebellar ataxias, HD is inherited in an autosomal dominant manner and caused by a CAG repeat expansion in the *HTT* gene, producing an abnormally long polyQ tract in Huntingtin (HTT) protein (Tabrizi et al, 2020). HTT is expressed ubiquitously, with high abundance in neurons, where it interacts with microtubule motors, endosomal adapters, and actin regulators to coordinate vesicular trafficking, axonal transport, organelle positioning, mitochondrial dynamics, and autophagy (Schulte and Littleton, 2011; Tabrizi et al, 2020). It also modulates transcriptional programs that support neuronal survival and energy homeostasis (Zuccato et al, 2003). In vulnerable neuronal populations, such as striatal medium spiny neurons, mutant HTT (mHTT) disrupts these pathways through both toxic gain- and loss-of-function effects, leading to defects in diverse HTT functions (Jiang et al, 2023; Tabrizi et al, 2020).

HD has been linked to the centrosome–cilium–satellite axis at multiple levels (Fig. 2). Wild-type HTT localizes to the centrosome, forms a complex with Huntingtin-associated protein 1 (HAP1) and the satellite scaffolding protein PCM1, and promotes Dynein-dependent pericentrosomal clustering of satellites, a process required for normal ciliogenesis (Aydin et al, 2020; Keryer et al, 2011; Liu and Zeitlin, 2011). Loss of HTT or HAP1 in striatal neurons disperses satellites and reduces cilium formation, while deletion in ependymal cells disrupts satellite organization, alters the ciliary layer, and causes hydrocephalus due to impaired CSF (Keryer et al, 2011). Similarly, *Xenopus* embryos lacking Httn (the Huntingtin orthologue) exhibit defective cilium polarity and neurogenesis (Haremaki et al, 2015). In contrast, polyQ-expanded Htt causes Pcm1 accumulation at the centrosome and leads to abnormally elongated cilia in striatal neurons, a phenotype also observed in HD mouse models and postmortem patient tissue (Keryer et al, 2011). In knock-in models expressing mHtt, primary cilium length is selectively increased in striatal but not cortical neurons, correlating with mHtt accumulation and altered dopamine receptor and mTOR signaling (Mustafa et al, 2019). Together, these findings identify an HTT-HAP1-PCM1 pathway that regulates satellite positioning and ciliogenesis, and show that mHTT alters ciliary organization in a cell type-specific manner in HD models and patient tissue. Because HTT also regulates vesicular trafficking, transcription, mitochondrial dynamics, and proteostasis, it remains unresolved whether these ciliary and satellite changes act as early contributors to striatal vulnerability or arise as downstream or adaptive consequences of broader mHTT-dependent cellular stress.

Beyond neurons, Htt localizes to both sensory and motile cilia across tissues, reflecting its broad role in ciliary structure and function (Yang et al, 2024). In photoreceptors, Htt loss results in elongated, hyperacetylated cilia and mislocalization of Ift57 and Ift88, which disrupts ciliary transport and causes accumulation of outer segment proteins in the cell body (Yang et al, 2024). These structural and trafficking defects compromise photoreceptor integrity and precede cell death, consistent with retinal pathology in HD mouse models (Helmlinger et al, 2002; Karam et al, 2015; Yang et al, 2024). These retinal phenotypes show that HTT-dependent ciliary trafficking defects can compromise long-term maintenance of specialized sensory neurons, although their relationship to striatal neurodegeneration in HD remains indirect.

Proteostasis failure is a hallmark of HD, and emerging evidence links this dysfunction to the centrosome–cilium–satellite axis. Satellite proteins such as PCM1 and CEP131, along with distal centriole components CP110, CEP97, and CEP290, promote the coalescence and growth of protein aggresomes, including mHTT aggregates, around centrioles (Prosser et al, 2022). Loss of these proteins disrupts aggresome formation and induces autophagy, indicating roles in balancing aggregate sequestration and clearance (Prosser et al, 2022). Reduced CP110 levels in senescent cells similarly impair aggresome formation, linking age-associated centrosomal changes to proteostasis decline in aging and NDDs (Prosser et al, 2022). These findings connect mHTT aggregation to centrosome- and satellite-associated proteostasis pathways, but the extent to which this contributes to selective striatal vulnerability remains to be determined.

In sum, HD provides one of the clearest classical NDD contexts in which centriolar satellites are mechanistically linked to disease-relevant ciliary phenotypes. The HTT-HAP1-PCM1 pathway connects satellite positioning to ciliogenesis, while mHTT-associated changes in ciliary length, ciliary trafficking, and centrosome-associated aggresome formation link this axis to both neuronal signaling and proteostasis. These findings suggest that mHTT perturbs the centrosome–cilium–satellite axis at multiple levels, but they should be interpreted alongside established HD mechanisms, including polyQ toxicity, transcriptional dysregulation, mitochondrial impairment, vesicular transport defects, and proteostasis failure. Future work should determine whether restoring satellite positioning or ciliary homeostasis can modify HD-relevant neuronal phenotypes independently of, or in combination with, broader effects on mHTT aggregation, trafficking, and mitochondrial function.

### Centriolar satellites in a related neurodevelopmental and neuropsychiatric context: schizophrenia

Schizophrenia (SZ) is a highly heterogeneous neuropsychiatric disorder with strong neurodevelopmental features (Gupta and Kulhara, 2010; Stone et al, 2022). Although SZ is not classified as a classical NDD, patient tissue, omics, and animal model studies have identified neurodegeneration-like cellular abnormalities, including mitochondrial dysfunction, oxidative stress, proteostasis defects, ER stress, and synaptic pathology (Howes and Onwordi, 2023; Nascimento and Martins-de-Souza, 2015; Nucifora et al, 2019; Stone et al, 2022). This overlap supports considering centrosome-, cilium-, and satellite-associated pathways in SZ. Consistent with this biological complexity, genome-wide association and sequencing studies reveal a complex genetic landscape in which numerous common and rare variants contribute modestly to disease risk (Sullivan et al, 2012). While genes involved in synaptic transmission are consistently implicated, they account for only part of SZ heritability, indicating that additional biological pathways contribute to disease pathophysiology (Howes and Onwordi, 2023; Purcell et al, 2014; Schizophrenia Working Group of the Psychiatric Genomics, 2014). Among these, the centrosome–cilium–satellite axis has emerged as relevant to selected SZ-related phenotypes, particularly those involving neuronal development, ciliary signaling, ventricular morphology, and dopaminergic regulation (Fig. 2) (Pruski and Lang, 2019).

Key signaling pathways implicated in SZ, particularly dopaminergic and serotonergic signaling, are closely linked to the primary cilium. Several neuromodulatory GPCRs localize to neuronal cilia, including D1-type dopamine receptors and serotonin 5-HT6 receptors, providing a potential mechanism through which ciliary dysfunction could affect pathways relevant to cognition, perception, and circuit regulation (Barbeito and Garcia-Gonzalo, 2021; Domire et al, 2011). Consistent with this possibility, patients with SZ exhibit fewer primary cilia in specific brain regions than healthy controls (Munoz-Estrada et al, 2018). Transcriptome-wide association studies further suggest altered expression of ciliary gene networks in SZ (Alhassen et al, 2021; Gandal et al, 2018). Genes encoding components of the transition zone, axoneme, BBSome, basal body, IFT machinery, and ciliary membrane are differentially expressed in SZ, suggesting that ciliary structure, trafficking, and signaling pathways may be altered in SZ-related neurodevelopmental biology (Alhassen et al, 2021).

Indeed, experimental and genetic studies converge on a role for cilia and centriolar satellites in SZ-related phenotypes. In *Pcm1* knockout mice, progressive postnatal loss of neuronal cilia integrity is accompanied by ventricular enlargement, a structural abnormality frequently observed in SZ that reflects changes in CSF and surrounding brain volume. These mice also exhibit cortical and striatal thinning, cognitive impairment, and altered dopaminergic signaling, including reduced D₂ receptor availability and resistance to antipsychotic treatment (Monroe et al, 2020). This antipsychotic resistance, also observed in patients carrying rare *PCM1* variants, suggests that ciliary dysfunction may contribute to treatment-resistant SZ by altering dopaminergic receptor regulation. Supporting this idea, zebrafish *pcm1* morphants show ventricular enlargement that can be rescued by wild-type human *PCM1* mRNA, whereas several SZ-associated patient variants fail to do so. This indicates that these patient-derived alleles are pathogenic and impair PCM1 function in vivo (Monroe et al, 2020). Ciliary defects can also affect dopamine signaling through other mechanisms. In mouse models of 22q11.2 deletion syndrome, haploinsufficiency of *Dgcr8*, a microRNA-processing gene within the deleted locus, elevates D1-receptor signaling, reduces ependymal ciliary motility, and causes ventricular enlargement (Eom et al, 2020). Together, these findings link satellite- and cilium-associated dysfunction to neuronal organization, ventricular phenotypes, and dopaminergic signaling in SZ-related neurodevelopmental models.

SZ-related defects in PCM1 extend to its interacting partners at satellites. Disrupted-in-schizophrenia 1 (*DISC1*), a well-established SZ risk gene involved in neurodevelopment, forms a complex with PCM1 and the ciliary protein BBS4 (Bradshaw and Porteous, 2012; Kamiya et al, 2008; Soares et al, 2011). In the developing cortex, suppression of *Pcm1* results in neuronal migration defects that are reproduced by loss of *Disc1* or *Bbs4* and become more severe when all three genes are suppressed together, highlighting their cooperative role in cortical development (Kamiya et al, 2008). Clinically, individuals with Bardet–Biedl syndrome, caused by mutations in *BBS4* and other *BBS* genes, show higher rates of cognitive deficits and psychosis than the general population, reinforcing the overlap between ciliopathies and psychiatric disease (Kerr et al, 2016). Another PCM1 interactor associated with both ciliopathies and SZ is SDCCAG8 (BBS16). It is a centrosome and transition-zone protein that regulates pericentrosomal clustering of satellites and is required for neuronal polarization and migration (Otto et al, 2010; Schaefer et al, 2011). Together, these studies identify a PCM1-DISC1-BBS4-SDCCAG8 network that links centriolar satellites to ciliary regulation, neuronal migration, ventricular morphology, and dopaminergic signaling. They also provide experimentally tractable satellite-related readouts, such as PCM1 organization, satellite positioning, and ciliary integrity, that can be tested in classical NDD models where satellite-specific mechanisms remain less well-characterized.

## Therapeutic strategies

**Figure 3:**
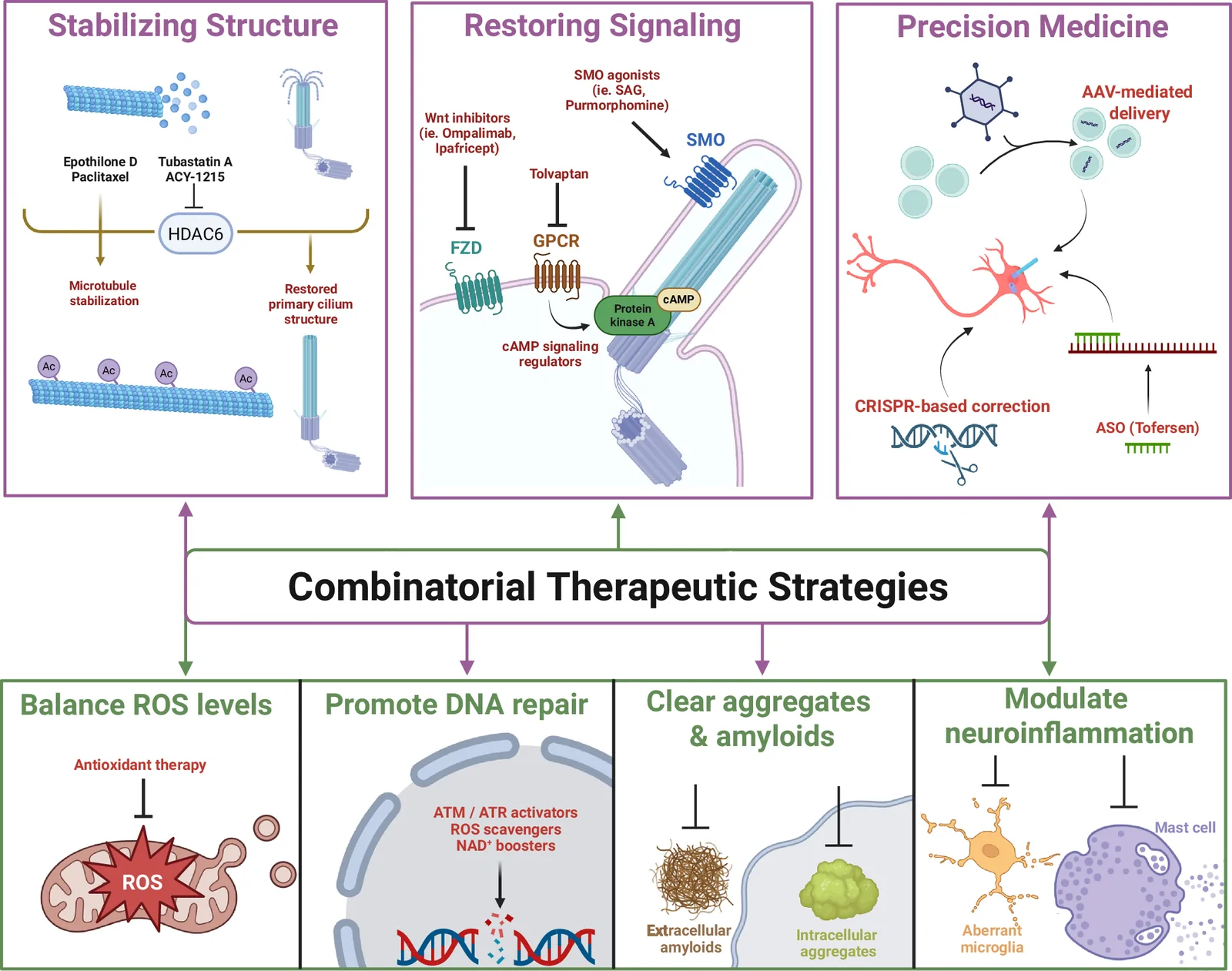
Therapeutic strategies for exploring centrosome–cilium–satellite axis dysfunction in disease-relevant models. The upper panel illustrates potential therapeutic entry points for modulating centrosome–cilium–satellite axis structure, signaling, and genetically defined defects. Stabilizing structure: microtubule stabilizers, including epothilone D and paclitaxel, promote microtubule stabilization, whereas HDAC6 inhibitors, including tubastatin A and ACY-1215, increase tubulin acetylation and may support restoration of primary cilium structure. Restoring signaling: small-molecule modulators target cilia-associated signaling pathways, including Wnt inhibitors acting through FZD, SMO agonists such as SAG and purmorphamine, tolvaptan-mediated GPCR/cAMP signaling modulation, and downstream cAMP/PKA signaling regulators. Precision medicine: AAV-mediated gene delivery, CRISPR-based gene correction, and antisense oligonucleotide approaches (e.g., tofersen) are designed to correct or compensate for mutations affecting centrosome–cilium–satellite biology. The lower panel illustrates how these approaches may be incorporated into broader combinatorial therapeutic strategies that target established NDD mechanisms. These include balancing ROS levels through antioxidant-based approaches; promoting DNA repair via ATM/ATR activators, ROS scavengers, or NAD⁺ boosters; clearing pathological protein species such as extracellular amyloids and intracellular aggregates; and reducing inflammation by modulating aberrant microglial or mast-cell activity. These strategies remain largely preclinical. The schematic illustrates a mechanism-based testing framework rather than established disease-modifying therapies for NDDs. Created in BioRender. Begar, E. (2026) https://BioRender.com/e1wdugh.

The centrosome–cilium–satellite axis provides potential entry points for therapeutic exploration because it intersects with cytoskeletal organization, ciliary signaling, vesicular trafficking, proteostasis, mitochondrial stress, and inflammatory pathways. However, therapeutic modulation of this axis in NDDs remains largely preclinical, and most interventions also influence broader cellular processes. We therefore discuss strategies for restoring axis structure, modulating ciliary signaling, correcting genetic defects, and prioritizing combinatorial approaches while emphasizing parallel assessment of ciliary and non-ciliary effects (Fig. 3).

### Restoring centrosome–cilium–satellite axis assembly and disassembly

A first therapeutic strategy is to restore the structural features of the centrosome–cilium–satellite axis, including microtubule stability, cilium maintenance, satellite positioning, and centrosome organization (Fig. 3). Because these processes depend on microtubules and their post-translational regulation, microtubule-associated pathways have emerged as a promising approach (Dogterom and Koenderink, 2019; Mirvis et al, 2018; Pedersen et al, 2012). A central target in this context is HDAC6, which regulates tubulin acetylation across cytoplasmic and axonemal microtubules and controls processes such as cilium disassembly, microtubule stability, and axonal transport in neurons (Benoy et al, 2017; Guo et al, 2017; Hubbert et al, 2002). Inhibition of HDAC6 with compounds such as tubastatin A, ACY-1215 (ricolinostat), and ACY-738 enhances tubulin acetylation and alleviates cytoskeletal and neuronal defects in models of ALS, AD, PD, and HTT (Benoy et al, 2017; Guo et al, 2017; Majid et al, 2015; Phipps et al, 2024). In many of these studies, however, benefit was assessed through broader neuronal phenotypes, including axonal transport, survival, or aggregate clearance, rather than through direct rescue of ciliary defects.

More direct preclinical evidence for cilium-related rescue comes from NEK1-linked ALS cellular models. In NEK1-linked ALS models, HDAC6 inhibition increased α-tubulin acetylation and partially rescued ciliary defects in patient-derived fibroblasts and iPSC-derived motor neurons, while also improving mitochondrial distribution/length defects in fibroblasts and apoptotic cell-death phenotypes in motor neurons (Noh et al, 2025). These findings make NEK1-linked ALS a particularly informative preclinical setting for testing whether restoration of ciliary stability can modify disease-relevant neuronal phenotypes. Related evidence from kidney ciliopathy models also supports the principle that HDAC6 inhibition can affect ciliary structure. HDAC6 inhibitors such as ACY-1215 lengthened cilia and slowed cyst progression in organoids and animal models (Bai et al, 2022; Cebotaru et al, 2016; Lorenzo Pisarello et al, 2018). Together, these studies provide pharmacological evidence for cilium-related structural rescue, although their relevance to NDDs requires validation in neural models, and they do not fully separate ciliary rescue from broader HDAC6-dependent effects.

Microtubule-stabilizing compounds provide a complementary strategy, especially in diseases where tau, α-synuclein, or mutant HTT alter microtubule dynamics and transport. Paclitaxel (Taxol), a well-characterized microtubule stabilizer, restored axonal transport and reduced motor deficits in tauopathy mouse models (Zhang et al, 2005). Although its poor blood-brain barrier penetration limits clinical use in neurological disorders, brain-penetrant analogs have been developed (Lou et al, 2014). Among these, epothilone D increased microtubule density, preserved axonal integrity, and improved cognitive and motor outcomes in models of AD and PD (Brunden et al, 2010; Cartelli et al, 2013; Guo et al, 2020). Unlike HDAC6 inhibitors, microtubule stabilizers do not primarily act by increasing tubulin acetylation; instead, they stabilize microtubule polymers and may indirectly influence cilium stability, intracellular trafficking, and neuronal architecture.

Together, HDAC6 inhibition and microtubule stabilization offer practical preclinical entry points for modulating axis structure and dynamics. Future studies should pair ciliary and centrosomal readouts with disease-relevant outcomes to distinguish cilium-specific rescue from broader cytoskeletal effects.

### Modulating ciliary signaling pathways

A second therapeutic strategy involves modulating cilium-associated signaling pathways whose activity is altered in specific NDD models (Fig. 3). Pathways such as Shh, Wnt, TGF-β, and ciliary GPCR/cAMP signaling are attractive therapeutic targets because they regulate neuronal development, homeostasis, survival, and stress responses. However, because these pathways have broad and context-dependent functions, therapeutic benefit will require disease- and cell type-specific modulation rather than uniform activation or inhibition.

Shh signaling illustrates this context dependence. In PD animal models, pharmacological activation of Shh signaling using Smo agonists such as SAG or purmorphamine protects dopaminergic neurons and attenuates inflammatory responses (Malave et al, 2021; Shao et al, 2017; Zhang et al, 2024). In contrast, PINK1-deficient and sporadic PD models show shortened primary cilia together with increased Shh signal transduction, and inhibition of this pathway restored ciliary morphology and mitochondrial function (Schmidt et al, 2022). In *SOD1*-G93A ALS mice, pharmacological activation of Shh signaling prolongs survival, whereas SHH inhibition shortens survival, further supporting the therapeutic relevance of Shh modulation in this model (Qi et al, 2022). Wnt signaling is another cilium-associated pathway with therapeutic potential, although evidence in NDD models remains limited. Wnt-pathway inhibitors, including FZD-targeting agents such as Ompalimab/vantictumab and the Wnt ligand-sequestering decoy receptor ipafricept, have been developed in other disease contexts and illustrate pharmacological strategies for dampening Wnt signaling (Davis et al, 2020; Dotan et al, 2020; Jung and Park, 2020). Their relevance to NDD-associated ciliary defects requires direct testing in neural models with matched ciliary and disease-level readouts. TGF-β signaling also shows why pathway modulation must be tailored to the disease context. TGF-β/Smad activation can reduce cytoplasmic TDP-43 aggregate formation in cell models, whereas astrocyte-derived TGF-β1 accelerates disease progression in *SOD1*-G93A mice, where pharmacological TGF-β inhibition extends survival (Endo et al, 2015; Nakamura et al, 2013). Together, these examples indicate that therapeutic modulation of cilium-associated signaling should first define pathway state, disease-relevant cell type, and intervention timing.

Ciliopathy research supports the feasibility of therapeutically modulating cilia-associated signaling. In Bardet–Biedl syndrome and polycystic kidney disease, drugs that regulate cAMP levels or GPCR trafficking improve cilia-mediated signaling and tissue function in preclinical and clinical studies (Carter et al, 2012; Devlin et al, 2023; Duong Phu et al, 2021; Kim et al, 2018). Tolvaptan, a vasopressin V2 receptor antagonist approved for autosomal dominant polycystic kidney disease (ADPKD), provides a clinical proof-of-principle for modulating cilia-associated cAMP signaling in ADPKD (Carraro et al, 2024; Devlin et al, 2023). Because cAMP signals through downstream effectors, including PKA, future studies should determine whether altered ciliary GPCR/cAMP signaling changes localized PKA activity and whether PKA could serve as a therapeutic target for restoring dysregulated cilium-dependent signaling in NDD models. However, these examples should not be interpreted as direct evidence for NDD treatment, because kidney ciliopathies and adult-onset NDDs differ in tissue context, disease timing, and pathogenic mechanisms. Therefore, signaling-based interventions in NDD models should be evaluated alongside ciliary readouts, such as receptor localization or pathway activity, and disease-relevant outcomes, including neuronal survival, synaptic function, mitochondrial state, and inflammatory responses.

### Precision medicine and gene therapy

A third therapeutic avenue involves precision medicine in genetically defined disease contexts (Fig. 3). Gene replacement and genome-editing strategies, including AAV-mediated delivery of functional proteins of the centrosome–cilium–satellite axis and CRISPR-based correction of pathogenic alleles, are emerging as powerful tools to restore cellular defects. However, their application to NDD-associated centrosome–cilium–satellite genes remains at an early stage and will depend on variant mechanism, target expression, delivery, and safety. Antisense oligonucleotides (ASOs) and siRNAs may further complement these approaches by modulating mutant transcripts, correcting splicing defects, or reducing toxic protein species. For toxic gain-of-function variants, allele-selective ASOs could in principle suppress mutant transcripts while preserving wild-type expression. In contrast, loss-of-function alleles may require gene augmentation, splice correction, or other approaches that restore functional protein. The clinical development of the *SOD1*-targeting ASO tofersen provides an important precedent for genetically targeted ASO therapy in ALS (Cerillo and Parmar, 2025; Georgiadou et al, 2026; Lauffer et al, 2024). As more NDD-associated mutations are identified, targeted correction, augmentation, splice correction, or allele-specific silencing strategies should be evaluated.

### Toward combinatorial treatments for NDDs

Because NDD pathogenesis involves multiple interacting cellular pathways, combination strategies may be more effective than single-target approaches for selected disease contexts. For the centrosome–cilium–satellite axis, this means pairing interventions that restore ciliary or centrosomal structure and signaling with treatments targeting established disease hallmarks, such as protein misfolding, mitochondrial dysfunction, neuroinflammation, cytoskeletal instability, impaired autophagy, oxidative stress, DNA damage, and defective repair responses (Fig. 3). Strategies that balance ROS levels, such as antioxidant therapy and ROS scavengers, may be particularly relevant when mitochondrial dysfunction and oxidative damage are prominent. Similarly, interventions that support DNA repair or NAD⁺ metabolism may complement axis-directed therapies in models where oxidative DNA damage, impaired ATM/ATR-dependent DNA damage responses, or age-associated metabolic decline contribute to neuronal vulnerability (Lautrup et al, 2019; Yan et al, 2014) (Fig. 3). For example, HDAC6 inhibition or microtubule stabilization could be combined with ciliary signaling modulators in models where cytoskeletal defects and altered ciliary signaling coexist, such as NEK1-linked ALS. Alternatively, HDAC6 inhibition could be paired with mitochondrial enhancers such as urolithin A or nicotinamide riboside to support energetic and redox balance, or with anti-inflammatory modulators to reduce microglial activation (Burtscher et al, 2022; Li et al, 2025; Madsen et al, 2024). Similarly, combining ciliary signaling modulators with drugs that promote autophagy or aggregate clearance, such as rapamycin analogs or trehalose, may simultaneously correct defects in signaling and proteostasis (Menzies et al, 2017; Sarkar et al, 2007). Non-axis-directed agents could also serve as complementary modules when they target dominant pathogenic processes in the same disease model; for example, masitinib may complement axis-directed strategies by modulating neuroinflammation in ALS or AD (Hermine et al, 2025), whereas Engrailed-1-based approaches may provide neuroprotective support in ALS or PD models (Leboeuf et al, 2023; Sonnier et al, 2007). Because neuroinflammation involves multiple immune cell types, including microglia and mast cells, combination strategies should also consider immune-cell interactions that contribute to blood-brain barrier disruption, cytokine release, and inflammatory amplification in neurodegenerative disease contexts (Jones et al, 2019).

Although direct evidence for therapeutic synergy in NDD models remains limited, ciliopathy models illustrate how complementary interventions can be combined rationally. In a polycystic liver disease model, combining the HDAC6 inhibitor ACY-1215 with the somatostatin receptor agonist pasireotide synergistically reduced liver and kidney cyst growth, increased primary cilium length, and lowered cAMP-dependent proliferative signaling (Lorenzo Pisarello et al, 2018). This supports the principle that interventions affecting ciliary structure and GPCR/cAMP signaling can cooperate in ciliopathy models. A related ADPKD study using a hypomorphic Pkd1 mouse model showed that combining tolvaptan with pasireotide produced an additive reduction in cyst progression, significantly reduced cystic and fibrotic volume, and decreased cAMP to wild-type levels (Hopp et al, 2015). These examples provide a rationale for testing analogous combinations in neuronal systems, but they should not be treated as direct evidence for NDD therapy because disease timing, cell type, and tissue physiology differ substantially between kidney/liver ciliopathies and adult-onset neurodegeneration.

Prioritizing combinatorial strategies should be guided by mechanism, model, and measurable rescue. First, combinations should be tested in disease models with a strong genetic or mechanistic link to the centrosome–cilium–satellite axis, such as NEK1/C21orf2-linked ALS, TTBK2-linked SCA11, LRRK2-dependent PD models, or HTT-HAP1-PCM1-dependent HD models. Second, efficacy should be assessed using matched axis-level and disease-level readouts. Axis-level readouts may include cilium length, ciliary receptor localization, Shh/Wnt/GPCR signaling, centrosome organization, and satellite distribution, whereas disease-level outcomes should include neuronal survival, synaptic function, axonal transport, mitochondrial state, aggregate burden, inflammatory output, ROS levels, DNA damage markers, DNA repair activity, and microglia–mast cell inflammatory interactions where relevant. Third, combinations should be evaluated for pathway interference and toxicity, because the targeted processes, including HDAC6, Shh, Wnt, TGF-β, LRRK2, autophagy, microtubule regulation, ROS control, DNA damage responses, NAD⁺ metabolism, and immune-cell modulation, have broad physiological roles. Risk mitigation should therefore include cell type-specific assays, dose-response studies, disease-stage-aware intervention windows, parallel assessment of ciliary and non-ciliary effects, and safety monitoring in tissues where the target has known physiological functions. Rather than treating centrosome–cilium–satellite modulation as a broadly disease-agnostic strategy, future work should use it to define mechanism-based combinations in genetically or phenotypically stratified NDD models.

## Conclusions and future directions

The evidence reviewed here supports a model in which centrosome–cilium–satellite alterations intersect with established mechanisms of neuronal vulnerability. These alterations are linked to classical NDD pathways, including proteostasis failure, cytoskeletal disruption, mitochondrial stress, altered signaling, and inflammation, and may both respond to and reshape these stress pathways. Their position in disease trajectories is likely context dependent: in selected genetic settings, centrosomal, ciliary, or satellite defects may act early, whereas in sporadic or aggregation-dominated NDDs they may function as modifiers, compensatory responses, or late-stage amplifiers. Defining these temporal and causal relationships will be important for patient stratification, biomarker development, and mechanism-based therapeutic design.

Clinical translation will require testing centrosome–cilium–satellite phenotypes in disease-appropriate models without assuming that the axis has the same causal role across disorders. Axis-level readouts, including ciliary structure, receptor localization, ciliary signaling, centrosome organization, and satellite distribution, should be paired with disease-relevant outcomes such as neuronal survival, synaptic function, axonal transport, mitochondrial state, aggregate burden, and inflammatory output. Compounds with preclinical or clinical-development experience, including HDAC6 inhibitors and microtubule stabilizers such as epothilones, can provide pharmacological entry points for testing whether modulation of cytoskeletal and ciliary pathways alters these phenotypes in neuronal and glial systems (Brunden et al, 2012; Shen and Kozikowski, 2020). In parallel, patient-derived iPSC models, brain organoids, super-resolution imaging, intravital imaging, and ciliopathy-derived approaches can help assess axis structure, function, and disease-relevant cellular phenotypes (Meyer et al, 2016; Shaikh et al, 2025). Small molecules, gene therapies, and signaling modulators developed for ciliopathies may also inform NDD studies, provided that their relevance is tested in neuronal and glial models rather than inferred directly from developmental or non-neural ciliopathy systems.

Recent spatial proteomic, proximity-labeling, super-resolution, and ultrastructural imaging studies are beginning to reveal cell type-specific diversity in centrosome and cilium composition, structure, and function. These differences likely reflect distinct physiological demands across neural cell types and may contribute to selective vulnerability in disease. Future work combining cell type-specific proteomics, high-resolution imaging, live signaling reporters, and multiomics approaches will be essential for linking axis structure and function to pathology across neural cell types, developmental stages, aging, and disease states.

Centriolar satellites represent a particularly important frontier. Their regulatory roles in centrosome and cilium biology are well-established, but their direct disease-specific roles in classical NDDs remain less extensively characterized than those of centrosomes and primary cilia. Future studies should therefore include satellite-specific readouts, such as PCM1 organization, satellite mobility, satellite proteome remodeling, local translation, autophagic turnover, and aggresome-associated functions. These analyses will be especially informative in contexts where satellite biology is already implicated, including Huntington’s disease and schizophrenia-related neurodevelopmental models.

Moving forward, the field should shift from descriptive disease phenotypes toward testable mechanisms, stratified model systems, and rational therapeutic combinations. Box 1 outlines key open questions that will be central to this effort, including timing, causality, cell type specificity, satellite biology, the relevance of ciliopathy mechanisms, and the use of axis-level phenotypes for therapeutic stratification.

Box 1 In need of answers

- **When and where do centrosome–cilium–satellite alterations matter during disease progression?**
  A major unresolved question is whether these alterations act as early contributors, disease modifiers, compensatory responses, or late-stage amplifiers. Addressing this will require longitudinal analyses in human tissue, patient-derived models, and animal systems across disease stages. It will also be important to distinguish stronger genetic or mechanistic contexts, such as NEK1/C21orf2-linked ALS, TTBK2/SCA11, LRRK2-dependent PD models, and HTT-HAP1-PCM1-dependent HD models, from more indirect or correlative associations.
- **Which disease phenotypes are axis-specific, and which reflect broader classical NDD pathways?**
  Many disease-linked proteins, including LRRK2, PINK1/Parkin, HTT, α-synuclein, C9orf72, TDP-43, and SOD1, are highly pleiotropic. Their centrosomal, ciliary, or satellite-associated functions overlap with classical NDD mechanisms such as proteostasis failure, mitochondrial dysfunction, lysosomal impairment, cytoskeletal disruption, RNA dysregulation, and stress responses. Defining the contribution of each layer will require separation-of-function mutants, targeted rescue experiments, and parallel assessment of axis-level and disease-level readouts.
- **Which neural cell types are most vulnerable to centrosome–cilium–satellite disruption?**
  Neurons, microglia, astrocytes, ependymal cells, Purkinje cells, motor neurons, striatal neurons, and photoreceptors may differ in axis organization and disease response. Cell type-specific proteomics, live imaging, spatial transcriptomics, and in situ proximity-labeling approaches are needed to define how centrosomes, cilia, and satellites vary across neural cell types, disease stages, and aging.
- **What are the disease-specific roles of centriolar satellites in classical NDDs?**
  Satellites regulate ciliogenesis, ciliary trafficking, autophagy, proteostasis, stress responses, and aggresome formation, but their direct roles in classical NDDs remain less characterized than those of centrosomes and primary cilia. Future studies should include satellite-specific readouts, such as PCM1 organization, satellite dynamics, satellite proteome remodeling, local translation, autophagic turnover, and aggresome-associated functions.
- **How should ciliopathy biology inform NDD models?**
  Ciliopathies demonstrate that centrosome–cilium dysfunction can disrupt brain development, ventricular homeostasis, CSF flow, retinal maintenance, and selected neural populations. However, developmental ciliopathies and adult-onset NDDs remain distinct disease contexts. iPSC-derived neural models, organoids, and animal models should be used to test which ciliopathy-derived mechanisms are relevant to adult neuronal vulnerability, aging, and degeneration.
- **Can centrosome–cilium–satellite phenotypes guide therapeutic stratification?**
  Therapeutic modulation of this axis in NDDs remains largely preclinical. Future studies should pair axis-level readouts, such as cilium length, receptor localization, ciliary signaling, centrosome organization, and satellite distribution, with disease-relevant outcomes, including neuronal survival, synaptic function, axonal transport, mitochondrial state, aggregate burden, and inflammatory output. This will be essential for prioritizing mechanism-based combinations and avoiding broad, disease-agnostic therapeutic claims.

## Supplementary information

Peer Review File
