# Supplementary material for: The centrosome-cilium-centriolar satellite axis in neurodegenerative diseases
Source: EMBO Rep. 2026 Jul 21;27(16):4624–52. doi: 10.1038/s44319-026-00877-3 (PMC13503701; doi:10.1038/s44319-026-00877-3)
Supplement: Supplementary file 1 [file 44319_2026_877_MOESM1_ESM.pdf]

EMBO reports

The centrosome-cilium-centriolar satellite axis in neurodegenerative diseases

Umut Sahin and Elif Firat-Karalar

Corresponding author(s): Elif Firat-Karalar (ekaralar@ku.edu.tr)

Review Timeline:

Submission Date:	18th Nov 25
Editorial Decision:	8th Jan 26
Revision Received:	21st May 26
Accepted:	18th Jun 26

Editor: Kurt Weir

Transaction Report:

(Note: With the exception of the correction of typographical or spelling errors that could be a source of ambiguity, letters and reports are not edited. Depending on transfer agreements, referee reports obtained elsewhere may or may not be included in this compilation. Referee reports are anonymous unless the Referee chooses to sign their reports.)

Dear Dr. Firat-Karalar

Thank you once more for the submission of your review to EMBO Reports. I have now received three referee reports that are copied below. As you will see, the referees state that your manuscript is interesting and timely, but they have also several suggestions to improve it, which I kindly ask you to address. In particular, bringing the perspective from the final sentence into the rest of the review. I would also like to highlight referee 1's comment on incorporating information on neurodegeneration in ciliopathies as a way to strengthen the paper.

Please also address the following editorial points:

- Please add a 'Disclosure and competing interests statement'. For more information see https://www.embopress.org/page/journal/14693178/authorguide#conflictsofinterest

- CRediT has replaced the traditional author contributions section because it offers a systematic machine readable author contributions format that allows for more effective research assessment. Please use the free text boxes beneath each contributing author's name in our online systems to add specific details on the author's contribution. More information is available in our guide to authors.

- Please carefully go through the reference list and make sure that each reference is complete with citation year, volume, and page/elocator numbers.
Please also adjust the format for citation of preprints as specified in our author guidelines:
The citation in the text should be: "(preprint: NAME1 et al, YEAR)"
The citation in the reference list: "Author NAME1, Author NAME2, ... (YEAR) article title. PLATFORM NAME, doi: XXX [PREPRINT]"
Please, refer to our detailed online guidelines for further information:
https://www.embopress.org/page/journal/14602075/authorguide#referencesformat

- When returning a revised version, may I also kindly ask you to include a box called "In need of answers" that briefly outlines the major questions that are still open in a given field in the form of a few bullet points. These questions can be accompanied by a brief explanation of what would be needed to address them and may prove helpful towards setting the stage for future experimentation in the field.

When submitting your revised manuscript, we will require a Microsoft Word file (.docx) of the revised manuscript text including detailed figure legends (at the very end), but without the figures.

- Please provide the final figures as separate, high resolution files as .pdf, .eps, .tif, or .jpg (one file per figure). Please finalize the drafts provided and make sure they accurately illustrate the key scientific concepts that you wish to show.

Please also note the following general advice on review figures:

- If there are certain aspects of your figure draft that are based upon assumptions or where the scientific data remains ambiguous (for example, schematically depicting a presumed direct protein-protein interaction, protein shape or subcellular localizations etc.) please add a comment so that we can work with you on an accurate depiction. Please ensure the directionality and nature of interactions is presented accurately.

- If the figure or single panels of the figure have been adapted from a published figure, please add this information to the figure legend (e.g., 'Adapted from...' or 'Based on...'). The editor will discuss if a reference and permission will be necessary

- Please only re-use figures or parts of a figure if this is essential for understanding the concept communicated. Often a reference to a previous paper will suffice. If the figure contains re-used images or elements of images, including schematics, micrographs or photos, please make sure that you have the permission/license to publish it (this also applies to your own previous work, if the journal you published in retains copyright. Certain 'creative commons' open access licenses, such as CC-BY 4.0, allow re-use without additional formal permissions). All re-used material must be explicitly cited.

- If you use an image data base for scientific iconography (e.g., BioRender), please let us know if you have a license that allows for publication in an academic journal. Often authors use misleading iconography for expedience. Please ensure the information shown is scientifically accurate. If in doubt, please discuss with the editor.

Thank you again for writing this article for us. I look forward to seeing a revised version of your manuscript when it is ready. As for timing, would it be possible for you to submit the revised version by end of March? But let me know if you need more time.

Best regards,

Kurt Weir
Editor
EMBO Reports

Referee #1:

The authors of this review postulate that dysfunction of the centrosome-cilium complex is a convergent mechanism for neurodegenerative diseases. While this a current, well written and interesting review makes an excellence case for centrosome-cilia dysfunction being an important driver of neurodegeneration I felt its importance, relative to other mechanisms, was potentially overstated. However, the wider role of dysfunction of the centrosome-cilium-satellite complex in neurodegenerative disease is fascinating and the authors do an excellent job of discussing this hypothesis. At times this review considers Schizophrenia and related conditions as neurodegenerative condition, while they may have a neurodegenerative component, I believe it would be better to discuss in terms of the centrosome-cilium complex's role in neurodevelopment.
Given the core hypothesis is that dysfunction of the centrosome-cilium complex is a convergent mechanism for neurodegenerative diseases the one area the review does not consider in depth is evidence for neurodegeneration (or neurodevelopmental abnormalities) in ciliopathies. If centrosome-cilia dysfunction is key for neurodegeneration is there evidence for this in ciliopathies? The authors could also explore whether there is any evidence for this the many models of ciliopathies. For example, the review mentions iPSC and organoid models in terms of drug testing, but what is known here already. This should be expanded in the review.
In summary, the review does an excellent job of articulating the role of of the centrosome-cilium complex in different neurodegenerative and neurodevelopmental conditions. The authors finishes with the sentence "With this perspective in mind, future studies should determine whether dysfunction of this axis [cilia : centrosome] constitutes an initiating trigger, a propagating driver, or a late-stage amplifier of neurodegenerative pathology" my main criticism is that this is caveat is not more prominent throughout the review.

Typo

Page 31 'In Bardet-Biedl syndrome and polycystic kidney disease, drugsthat regulate cAMP levels or GPCR trafficking' space between drugs and that.

Referee #2:

This review attempts to reframe neurodegenerative and psychiatric diseases as manifestations of a shared organelle vulnerability. The central claim is that dysfunction of the centrosome-cilium-centriolar satellite complex represents a unifying mechanism that connects classical hallmarks of neurodegeneration and provides a new conceptual framework across AD, PD, ALS, HD, hereditary ataxias and schizophrenia. The manuscript provides an up-to-date overview of ciliary and centrosomal biology and offers several illustrative examples of genes and pathways involved in ciliogenesis and ciliary signaling.

However, the manuscript suffers from conceptual and methodological shortcomings that prevent it from functioning as a balanced or evidence-based review. Most critically, mechanistic proximity is repeatedly treated as mechanistic primacy, with co-localization or signaling interactions implicitly elevated to causal pathways. Alternative and often more strongly supported mechanisms in neurodegeneration such as synaptic failure, immune activation, axonal and lysosomal dysfunction, metabolic vulnerability, are minimized whenever they cannot be assimilated into the ciliary framework. There is no attempt to define boundary conditions or falsification criteria, and the manuscript applies no hierarchy of evidence. For instance, in vitro perturbations, mouse models and rare familial mutations are treated as equivalent to human sporadic disease genetics.

The claim of a "unifying mechanism" also collapses fundamental disease heterogeneity. The authors acknowledge diversity in clinical and genetic forms of neurodegeneration but immediately reduce it to a single organellar axis, framed as a hallmark. The data support a role for this complex in particular disorders and cell types, but do not justify extending the model across the entire spectrum of diseases cited. The treatment of Alzheimer's disease is a clear example: mechanisms are extrapolated from familial APP/PSEN models to sporadic AD despite the absence of ciliogenesis genes in large-scale GWAS, and without considering the possibility that ciliary changes are downstream of Aβ, tau or inflammatory pathology.

Several major interpretive issues remain unresolved. The review never distinguishes whether ciliary changes are initiating, compensatory or terminal phenomena, despite reporting opposite phenotypes (elongation vs. shortening) within and across models. Many highlighted proteins (LRRK2, PINK1, HTT, α-synuclein) are strongly pleiotropic, yet the manuscript does not ask how much of the pathology is mediated through ciliary functions versus other well-established pathways. Even in HD and ciliopathy-linked schizophrenia, where the evidence is strongest, alternative mechanisms and intra-disease heterogeneity are not addressed. The therapeutic section inherits these weaknesses: cross-disease strategies are proposed without human data showing that modulating this complex alters disease independently of broader cytoskeletal or proteostatic effects.

Another major drawback is that the manuscript reveals a limited clinical and translational perspective. Complex, heterogeneous clinical entities are treated as if they can be collapsed into a single framework, with little regard for disease staging, selective vulnerability or real-world pathophysiology. This reductionist treatment of intrinsically heterogeneous disorders suggests a limited appreciation of clinical and translational complexity, and overestimates the explanatory reach of a single mechanistic narrative.

Finally, several recent reviews already consider centrosomes and primary cilia in neurodegeneration, signal caution about over-generalization, and highlight major gaps in causal evidence. So, the ambition to cast a universal cilium/centrosome-based mechanism over the full spectrum of neurodegenerative and psychiatric disorders is not as novel as implied. For example, Lattao 2025 (https://doi.org/10.1098/rsob.240317) argues that although many disease-linked proteins localize to centrosomes or cilia, there remains little direct evidence that these organelles are central drivers of neurodegeneration. Takahashi 2025 (https://doi.org/10.3389/fnins.2025.1688839) claims that ciliary involvement in ALS remains preliminary and calls for much more rigorous validation before considering cilia as a foundational pathogenic axis. These reviews do not appear to be cited in the manuscript, whereas they treat cilia and centrosomes as possible contributors but not as a unified paradigm for neurodegeneration. That context renders the present manuscript's claims of a "new hallmark" and a universal organelle-based mechanism overly ambitious and insufficiently grounded in the existing literature.

In summary, this work is most useful as an introduction to centrosome-cilium biology. However, it does not provide a rigorous or balanced evaluation of the wider neurodegeneration field, and its central thesis is insufficiently supported by human genetics, disease heterogeneity or mechanistic clarity. The extent of speculation and conceptual overreach means the manuscript reads more like a perspective or opinion piece advocating a unifying hypothesis than an evidence-based review. In my judgement, this makes it unsuitable for publication in EMBO reports. I do not recommend acceptance.

Referee #3:

The authors provide a revision of the current evidence identifying the cilium-centrosome-satellite axis as a possible unifying mechanisms to explain the cellular processes underlying neurodegeneration. I liked the general idea. However, that the final goal of describing the function of satellites in neurodegenerative disorders has not been reached. I found the review dispersive and not clear for what concerns the molecular aspects.

1. The term 'complex' typically refers to a physical assembly of proteins that interact directly to form a defined structural unit. In contrast, the centrosome-cilium-centriolar satellites system does not represent a single physical entity but rather a functional and spatial continuum. Therefore, referring to it as an 'axis' is more appropriate than complex.
2. The composition of centriolar satellites should be more clearly explained, as this is fundamental to understanding their scaffolding and regulatory functions. Expanding on how centriolar satellites contribute to the regulation of ciliogenesis through autophagic pathways would strengthen the narrative and provide a more comprehensive view of their functional significance. Clarifying this interplay would help readers better understand the mechanistic connections underlying satellite-mediated control of cilia formation.
3. The manuscript could provide a more consistent explanation of the inheritance patterns for all the diseases discussed. In some cases, such as the spinocerebellar ataxias (SCAs), the genetic inheritance is clearly described, whereas for other disorders the inheritance patterns are not explained in sufficient detail. Including this information throughout would improve clarity and help readers better understand the genetic basis of the diseases.
4. Page 5: 'Beyond its MTOC role, the centrosome also acts as a signaling hub by concentrating regulatory molecules involved in processes cell cycle control, DNA damage response, and other pathways 16. Structurally, the centrosome consists of two centrioles surrounded by the pericentriolar material (PCM), which contains microtubule- nucleating factors such as gamma-tubulin complex that confer the centrosome's MTOC activity. Centrioles are microtubule-based cylindrical structures that are structurally and functionally distinct, with the mother centriole has distal and subdistal appendages, whereas the daughter centriole lacks them.' (This section contains several wording and grammatical issues that should be corrected for clarity. Revising these points would improve readability)
5. Motile cilia are mentioned without prior introduction or proper context within the framework of neurodegenerative diseases. Providing a brief explanation of their relevance would help complement the topic integrating with some other literature material such as Ma, X. Y., Yang, T. T., Liu, L., Peng, X. C., Qian, F., & Tang, F. R. (2023). Ependyma in Neurodegenerative Diseases, Radiation-Induced Brain Injury and as a Therapeutic Target for Neurotrophic Factors. Biomolecules, 13(5), 754. https://doi.org/10.3390/biom13050754)
6. It would be helpful to review the gene nomenclature throughout the text, as it is often inconsistent or incorrect.
7. Page 25: 'Beyond its mitotic function, Ataxin-10 interacts with ciliary transition zone and basal body proteins, including NPHP5 and ARL13B, both critical for ciliogenesis.' (Listing ARL13B with basal body proteins may be misleading, as it primarily localizes to the ciliary membrane and axoneme. Clarification of its role in ciliogenesis is recommended.)
8. The manuscript highlights multi-target approaches to restore both ciliary and classical neurodegenerative pathways, which is a strong conceptual advance. However, the discussion remains largely descriptive. The authors should provide more concrete examples or models of combinatorial interventions, including evidence for synergy, potential risks, and strategies to mitigate off-target effects. Clarifying how these approaches could be prioritized for preclinical or clinical testing would strengthen the

translational impact of the review.
9. There are several elements in Figure 1 that need revision. In panel A, the arrow connecting Dynein-2 to the structure is projected onto the axoneme rather than the protein itself. Additionally, placing PCM1 in parentheses may be misleading, as it suggests that the structure consists solely of this protein. It would be more appropriate to indicate the Distal Appendages and Sub-distal Appendages in panel A rather than panel B. In panel B, near number 1, the disc that initiates recruitment of the proteins responsible for blocking elongation is not indicated. Finally, points 4 and 5 could be depicted more clearly to better illustrate the disassembly process.

Response to Reviewers

We thank the reviewers for their careful evaluation of our manuscript and for their constructive suggestions. We have substantially revised the review in response. The major changes include removal of the “unifying mechanism” and “new hallmark” framing, replacement of “complex” with “axis” where appropriate, stronger discussion of established NDD mechanisms, addition of a new ciliopathy section, clearer separation of schizophrenia from classical NDDs, expansion of the centriolar satellite component, revised therapeutic and Conclusions/Future Directions sections, addition of Box 1 (“In need of answers”) to summarize the major open questions and experimental priorities in the field, and updated figures and figure legends to reflect the new framework.

Referee #1:

1- The authors of this review postulate that dysfunction of the centrosome-cilium complex is a convergent mechanism for neurodegenerative diseases. While this a current, well written and interesting review makes an excellence case for centrosome-cilia dysfunction being an important driver of neurodegeneration I felt its importance, relative to other mechanisms, was potentially overstated. However, the wider role of dysfunction of the centrosome-cilium-satellite complex in neurodegenerative disease is fascinating and the authors do an excellent job of discussing this hypothesis.

We thank the reviewer for this important point and for the positive assessment of the overall concept. We agree that the original framing could be interpreted as assigning excessive causal weight to centrosome-cilium-satellite dysfunction relative to established mechanisms of neurodegeneration. We have therefore revised the manuscript throughout to avoid presenting this axis as a universal, singular, or primary driver of NDDs, or as a convergent mechanism that supersedes established disease pathways.

In the revised manuscript, we frame the centrosome-cilium-satellite axis as an emerging, context-dependent contributor to neuronal vulnerability that intersects with classical hallmarks, including proteostasis failure, cytoskeletal disruption, mitochondrial dysfunction, inflammation, synaptic pathology, and DNA/RNA dysregulation. We also strengthened the discussion of classical NDD hallmarks in the Introduction to emphasize that these remain the foundation for understanding neurodegenerative mechanisms.

We further revised the manuscript to make the strength and interpretation of the evidence more explicit. Rather than treating all observations as equivalent, we now highlight, where the literature allows, the type of evidence supporting each disease connection, including direct genetic links, patient-derived cellular models, animal models, and observations from human tissue. We also added disease-specific interpretive statements to clarify that centrosome-cilium-satellite dysfunction may occupy different positions in disease trajectories: in selected genetic contexts it may act early, whereas in other settings it may modify established pathology, reflect adaptive or compensatory responses, or amplify late-stage degeneration.

This revised framing is applied throughout the disease sections. For example, we now distinguish familial APP/PSEN- and Aβ-driven models from sporadic AD; clarify in the PD and HD sections that LRRK2, PINK1/Parkin, α-synuclein, and HTT have broad non-ciliary functions; and emphasize that evidence strength varies across diseases, cell types, and disease stages. We also revised the therapeutic and Conclusions/Future Directions sections to avoid broad cross-disease claims and instead emphasize mechanism-based, genetically or phenotypically stratified testing. In addition, we added Box 1 ("In need of answers") to highlight unresolved questions about timing, causality, cell type specificity, satellite biology, ciliopathy relevance, and therapeutic stratification.

2- At times this review considers Schizophrenia and related conditions as neurodegenerative condition, while they may have a neurodegenerative component, I believe it would be better to discuss in terms of the centrosome-cilium complex's role in neurodevelopment.

We thank the reviewer for this helpful suggestion. We agree that schizophrenia should not be presented as a classical NDD. We have therefore revised the manuscript to clearly separate schizophrenia from the main neurodegenerative disease framework. Schizophrenia is now described as a highly heterogeneous neuropsychiatric disorder in which neurodevelopmental mechanisms are central, while selected neurodegeneration-like cellular abnormalities are acknowledged where relevant.

We retained a focused schizophrenia section because it provides particularly informative evidence for centrosome-cilium-satellite biology in nervous-system development and function. The revised section emphasizes PCM1, DISC1, BBSome-related pathways, SDCCAG8, ciliary integrity, neuronal migration, ventricular morphology, and dopaminergic signaling. This also strengthens the satellite component of the review, since centriolar satellites are important regulators of centrosome organization, ciliogenesis, ciliary trafficking, and proteostasis, but their direct roles in classical NDDs remain less extensively characterized than those of centrosomes and primary cilia.

3- Given the core hypothesis is that dysfunction of the centrosome-cilium complex is a convergent mechanism for neurodegenerative diseases the one area the review does not consider in depth is evidence for neurodegeneration (or neurodevelopmental abnormalities) in ciliopathies. If centrosome-cilia dysfunction is key for neurodegeneration is there evidence for this in ciliopathies? The authors could also explore whether there is any evidence for this the many models of ciliopathies. For example, the review mentions iPSC and organoid models in terms of drug testing, but what is known here already. This should be expanded in the review.

We thank the reviewer for this helpful suggestion. We agree that ciliopathies provide an important reference point for evaluating the relevance of centrosome-cilium dysfunction in the nervous system. We have therefore added a new section entitled "Ciliopathies as models of centrosome-cilium dysfunction in the nervous system", positioned after the neuronal adaptations section and before the disease-specific NDD sections.

In this section, we now discuss neurological manifestations of ciliopathies, including Joubert syndrome, Bardet-Biedl syndrome, hydrocephalus, cerebellar and brainstem abnormalities, retinal degeneration, cognitive impairment, and behavioral phenotypes. We also added discussion of ependymal motile cilia and CSF flow, linking ciliary dysfunction to ventricular homeostasis and brain waste-clearance pathways relevant to NDD biology.

We further expanded the discussion of animal, iPSC-derived neural, and brain organoid models. These systems show how centrosome- and cilium-dependent pathways influence neural progenitor behavior, tissue organization, ventricular architecture, neuronal differentiation, and selected maintenance or degenerative phenotypes, such as retinal degeneration. At the same time, we now clarify that ciliopathies are most informative as models of neurodevelopmental and tissue-organization defects, with some neural maintenance phenotypes, and do not by themselves establish centrosome-cilium dysfunction as a universal cause of adult-onset NDDs. We therefore use ciliopathy biology to inform interpretation of centrosome-cilium defects in NDD models, while evaluating NDD-associated evidence within its own genetic, cellular, pathological, and disease-stage-specific context.

4- In summary, the review does an excellent job of articulating the role of of the centrosome-cilium complex in different neurodegenerative and neurodevelopmental conditions. The authors finishes with the sentence "With this perspective in mind, future studies should determine whether dysfunction of this axis [cilia : centrosome] constitutes an initiating trigger, a propagating driver, or a late-stage amplifier of neurodegenerative pathology" my main criticism is that this is caveat is not more prominent throughout the review.

We thank the reviewer for recognizing that the manuscript makes a strong case for the relevance of centrosome-cilium biology in neurodegenerative and neurodevelopmental conditions. We agree that the original version placed the key caveat too late in the manuscript, and we have now made this point central to the revised framing.

Specifically, we revised the title, abstract, Introduction, disease sections, therapeutic section, and Conclusions/Future Directions to avoid presenting centrosome-cilium-satellite dysfunction as a universal or primary mechanism. Instead, we now emphasize throughout that its role may differ by disease, genetic background, cell type, and disease stage, and may reflect an early contributor, disease modifier, compensatory response, or late-stage amplifier depending on context. We also added Box 1 to make these open questions explicit and to outline experimental approaches needed to resolve them.

We also added disease-specific interpretive statements where the available evidence allows. For example, we now distinguish familial APP/PSEN- and Aβ-driven AD models from sporadic AD; clarify that LRRK2, PINK1/Parkin, HTT, and α-synuclein have broad non-ciliary functions; identify NEK1/C21orf2-linked ALS and TTBK2/SCA11 as stronger genetic contexts for centrosome-cilium involvement; and frame HD as one of the clearer contexts for satellite-associated ciliary dysfunction while still emphasizing established HD

mechanisms. These changes make the “initiating trigger, propagating driver, or late-stage amplifier” caveat a guiding principle throughout the manuscript rather than a concluding qualification.

Typo: Page 31 'In Bardet-Biedl syndrome and polycystic kidney disease, drugsthat regulate cAMP levels or GPCR trafficking' space between drugs and that.

We have corrected this typo.

Referee #2:

This review attempts to reframe neurodegenerative and psychiatric diseases as manifestations of a shared organelle vulnerability. The central claim is that dysfunction of the centrosome-cilium-centriolar satellite complex represents a unifying mechanism that connects classical hallmarks of neurodegeneration and provides a new conceptual framework across AD, PD, ALS, HD, hereditary ataxias and schizophrenia. The manuscript provides an up-to-date overview of ciliary and centrosomal biology and offers several illustrative examples of genes and pathways involved in ciliogenesis and ciliary signaling.

We thank the reviewer for the detailed and critical assessment of the manuscript. We recognize that the original framing, particularly the use of terms such as “unifying mechanism” and “new hallmark,” could be interpreted as assigning excessive causal weight to centrosome-cilium-satellite dysfunction. We have therefore substantially revised the manuscript to make the conceptual framework more balanced, evidence-based, and disease-context specific.

In the revised manuscript, we no longer present centrosome-cilium-satellite dysfunction as a universal mechanism or primary cause of NDDs. Instead, we frame the centrosome-cilium-satellite axis as an emerging, context-dependent organelle system that intersects with established neurodegenerative mechanisms, including proteostasis failure, cytoskeletal and axonal dysfunction, mitochondrial impairment, lysosomal-autophagic defects, inflammation, synaptic pathology, and DNA/RNA dysregulation.

We have also revised the treatment of schizophrenia. It is no longer presented as a classical NDD, but is discussed separately as a neurodevelopmental and neuropsychiatric context that provides particularly informative evidence for satellite-associated ciliary biology.

However, the manuscript suffers from conceptual and methodological shortcomings that prevent it from functioning as a balanced or evidence-based review. Most critically, mechanistic proximity is repeatedly treated as mechanistic primacy, with co-localization or signaling interactions implicitly elevated to causal pathways. Alternative and often more strongly supported mechanisms in neurodegeneration such as synaptic failure, immune activation, axonal and lysosomal dysfunction, metabolic vulnerability, are minimized whenever they cannot be assimilated into the ciliary framework. There is no attempt to

define boundary conditions or falsification criteria, and the manuscript applies no hierarchy of evidence. For instance, in vitro perturbations, mouse models and rare familial mutations are treated as equivalent to human sporadic disease genetics.

We agree that the original manuscript did not make this distinction sufficiently explicit. We have revised the manuscript throughout to avoid equating localization, pathway interaction, or model-based perturbation with causality. Where appropriate, we now use more precise language such as “intersects with,” “is associated with,” “may contribute,” “may modify,” or “requires functional validation,” depending on the strength of evidence.

We also strengthened the “Classical hallmarks of neurodegenerative diseases” section to emphasize that established mechanisms, including protein aggregation, impaired proteostasis, axonal and cytoskeletal defects, mitochondrial dysfunction, lysosomal-autophagic impairment, inflammation, synaptic dysfunction, and nucleic acid defects, remain the foundation for interpreting NDD pathogenesis. Centrosome-cilium-satellite dysfunction is now presented as an organelle-level phenotype that intersects with these mechanisms, rather than as replacing them.

We also revised the disease sections to reflect that this is an emerging field in which the strength of evidence varies substantially across diseases and genes. Where the literature allows, we now identify the type of evidence supporting each connection, including direct genetic links, patient-derived cellular models, animal models, and human tissue observations. We emphasize that direct evidence for centrosome-cilium-satellite dysfunction is strongest in defined genetic or model contexts, such as NEK1/C21orf2-linked ALS, TTBK2/SCA11, LRRK2-dependent PD models, and HTT-HAP1-PCM1-dependent HD models. For many other NDD contexts, the evidence remains more indirect, correlative, or model-dependent.

For example, the revised AD section now distinguishes familial APP/PSEN- and Aβ-driven models from sporadic AD and explicitly considers that ciliary changes may arise downstream of amyloid, tau, aging-related stress, or neuroinflammation. The PD section now emphasizes that LRRK2, PINK1/Parkin, and α-synuclein have broad non-ciliary functions and that ciliary phenotypes vary by genotype, cell type, model system, and disease stage. The ALS section distinguishes relatively direct NEK1/C21orf2-linked centrosome-cilium evidence from more context-dependent C9orf72-, TDP-43-, and SOD1-associated phenotypes. Together, these revisions directly address the concern that mechanistic proximity was previously treated as mechanistic primacy. Box 1 further reinforces this evidence-based framing by identifying which questions require direct genetic, patient-derived, animal-model, human tissue, and functional validation approaches.

The claim of a "unifying mechanism" also collapses fundamental disease heterogeneity. The authors acknowledge diversity in clinical and genetic forms of neurodegeneration but immediately reduce it to a single organellar axis, framed as a hallmark. The data support a role for this complex in particular disorders and cell types, but do not justify extending the model across the entire spectrum of diseases cited. The treatment of Alzheimer's

disease is a clear example: mechanisms are extrapolated from familial APP/PSEN models to sporadic AD despite the absence of ciliogenesis genes in large-scale GWAS, and without considering the possibility that ciliary changes are downstream of Aβ, tau or inflammatory pathology.

We agree that the original wording was too broad. We have removed the "unifying mechanism" and "new hallmark" framing from the title, abstract, introduction, disease sections, therapeutic section, and Conclusions/Future Directions. The manuscript now emphasizes that the contribution of the centrosome-cilium-satellite axis varies by disease, genetic background, cell type, disease stage, and component of the axis.

The revised abstract now states that centrosome-cilium-satellite dysfunction intersects with established pathways in disease- and cell type-specific contexts, rather than defining a universal mechanism. We also added an introductory framing section explaining that centrosomal and ciliary evidence is stronger in some disease contexts than others, while satellite-specific mechanisms remain less extensively characterized in classical NDDs and are clearest in selected contexts such as Huntington's disease and schizophrenia-related neurodevelopmental models.

The treatment of Alzheimer's disease is a clear example: mechanisms are extrapolated from familial APP/PSEN models to sporadic AD despite the absence of ciliogenesis genes in large-scale GWAS, and without considering the possibility that ciliary changes are downstream of Aβ, tau or inflammatory pathology.

We have revised the AD section to address this concern. The revised section now explicitly distinguishes late-onset sporadic AD from rare autosomal-dominant familial forms involving APP, PSEN1, and PSEN2. We also clarify that much of the centrosome/cilium evidence in AD comes from APP/PSEN-based models, Aβ exposure systems, and amyloid-driven cellular and animal models.

We now state that ciliary defects in familial APP/PSEN-based and Aβ-driven systems may contribute to altered signaling, extracellular proteostasis, and inflammatory responses, but that in sporadic AD they may also arise downstream of amyloid, tau, aging-related stress, or neuroinflammation. To directly address the reviewer's point about human genetics, we added that large-scale sporadic AD genetic studies have not yet established core ciliogenesis pathways as a major risk category. We also revised the interpretation of opposing ciliary length phenotypes, such as shortening versus elongation, as context-dependent disruption of ciliary homeostasis rather than as a single uniform AD phenotype.

Together, these changes prevent extrapolation from familial or model-based AD systems to sporadic AD and make clear that centrosome-cilium dysfunction in AD is best viewed as an emerging, context-dependent phenotype that intersects with established AD mechanisms, rather than as a primary driver of disease.

Several major interpretive issues remain unresolved. The review never distinguishes whether ciliary changes are initiating, compensatory or terminal phenomena, despite reporting opposite phenotypes (elongation vs. shortening) within and across models.

We have made this issue central to the revised framework. The manuscript now states early on that centrosome-cilium-satellite dysfunction may occupy different positions in disease trajectories. In selected genetic contexts, such as NEK1/C21orf2-linked ALS or TTBK2/SCA11, defects may act early or as sensitizing factors. In other settings, such as sporadic AD, α-synuclein-linked PD models, or aggregation-dominated diseases, centrosomal or ciliary changes may instead modify established pathology, reflect adaptive remodeling, or amplify late-stage degeneration.

This framework is now applied throughout the disease sections. For example, the AD section interprets ciliary shortening and elongation as context-dependent homeostatic disruption. The PD section emphasizes that ciliary phenotypes vary by genotype, neuronal subtype, model system, and disease stage. The ALS section distinguishes relatively direct NEK1/C21orf2 links from more context-dependent C9orf72, TDP-43, and SOD1-associated phenotypes. The SCA section identifies TTBK2/SCA11 as the strongest direct ciliogenesis example while treating other ataxins as subtype-specific links.

Many highlighted proteins (LRRK2, PINK1, HTT, α-synuclein) are strongly pleiotropic, yet the manuscript does not ask how much of the pathology is mediated through ciliary functions versus other well-established pathways. Even in HD and ciliopathy-linked schizophrenia, where the evidence is strongest, alternative mechanisms and intra-disease heterogeneity are not addressed.

We agree that this point required clearer treatment. We have revised the disease sections to avoid implying that pleiotropic disease proteins act primarily through centrosome-cilium-satellite mechanisms.

In the PD section, we now discuss LRRK2, PINK1/Parkin, and α-synuclein in relation to their broader roles in mitochondrial quality control, lysosomal and vesicular trafficking, proteostasis, and α-synuclein aggregation. We also clarify that the strongest PD evidence concerns centrosome-cilium regulation rather than satellite-specific mechanisms, and that ciliary phenotypes vary by genotype, cell type, model system, and disease stage.

In HD, we highlight the HTT-HAP1-PCM1 pathway as a strong satellite-linked example, while emphasizing that these findings must be interpreted alongside established HD mechanisms, including polyQ toxicity, transcriptional dysregulation, mitochondrial impairment, vesicular transport defects, and proteostasis failure. In schizophrenia, we now frame the disorder as a neurodevelopmental and neuropsychiatric context and discuss PCM1-, DISC1-, BBS4-, and SDCCAG8-associated mechanisms as satellite/ciliary contributions to neuronal organization, ventricular morphology, and dopaminergic signaling rather than as a complete explanation of schizophrenia pathophysiology.

Thus, the revised manuscript presents centrosome-cilium-satellite dysfunction as one disease-relevant layer within broader pathogenic networks, with its contribution varying by disease, genetic background, cell type, and disease stage.

The therapeutic section inherits these weaknesses: cross-disease strategies are proposed without human data showing that modulating this complex alters disease independently of broader cytoskeletal or proteostatic effects.

We have revised the therapeutic section to make it more evidence-based and translationally cautious. We now state explicitly that therapeutic modulation of the centrosome-cilium-satellite axis in NDDs remains largely preclinical, and that there is currently no human evidence showing that selective modulation of this axis alters disease progression independently of broader cytoskeletal, proteostatic, mitochondrial, vesicular-trafficking, or inflammatory effects.

We no longer present centrosome-cilium-satellite modulation as a broadly disease-agnostic therapeutic strategy. Instead, we frame therapeutic opportunities around mechanism-based and genetically or phenotypically stratified testing. We added model-specific examples, including HDAC6 inhibition in NEK1-linked ALS models, where ciliary and microtubule-acetylation defects are partially rescued in patient-derived fibroblast and iPSC-derived motor neuron systems, and LRRK2-dependent PD models, where LRRK2/Rab-dependent defects in ciliogenesis and ciliary signaling can be modulated in preclinical systems. We emphasize that these findings remain preclinical and do not establish disease-modifying efficacy in humans.

We also added a dedicated discussion of combinatorial therapy and risk mitigation. Because direct evidence for therapeutic synergy in NDD models remains limited, we now use ciliopathy studies only as proof-of-concept examples for rational combination design, not as direct evidence for NDD treatment. We also added prioritization criteria, including cell type-specific assays, dose-response studies, disease-stage-aware intervention windows, parallel assessment of ciliary and non-ciliary effects, and safety monitoring in relevant tissues.

We further revised Figure 3 and its legend to match this cautious translational framing. The figure is now presented as a preclinical, mechanism-based testing framework rather than as a set of established disease-modifying therapies for NDDs. We also added Box 1 to identify therapeutic stratification as an open question and to emphasize that axis-level readouts should be paired with disease-relevant outcomes in appropriate neuronal and glial models.

Another major drawback is that the manuscript reveals a limited clinical and translational perspective. Complex, heterogeneous clinical entities are treated as if they can be collapsed into a single framework, with little regard for disease staging, selective vulnerability or real-world pathophysiology. This reductionist treatment of intrinsically

heterogeneous disorders suggests a limited appreciation of clinical and translational complexity, and overestimates the explanatory reach of a single mechanistic narrative.

We have revised the manuscript to better address disease heterogeneity, selective vulnerability, disease staging, and translational complexity. We now make clear that we do not present the centrosome-cilium-satellite axis as a single unifying explanation for all NDDs, but rather as a context-dependent cellular system whose role may vary by disease type, genetic background, affected cell population, and disease stage.

The Introduction now emphasizes that NDDs are complex and multifactorial, with dominant pathogenic mechanisms differing across clinical entities and disease trajectories. The disease-specific sections now include more explicit discussion of cell type-specific and stage-dependent phenotypes, including neuronal, microglial, and ependymal ciliary changes in AD models; striatal and cell type-specific phenotypes in PD and HD; motor neuron vulnerability in ALS; and Purkinje-cell vulnerability in SCA11.

We also revised the Conclusions/Future Directions and added Box 1 to emphasize approaches that better capture this complexity, including cell type-specific proteomics, high-resolution imaging, live signaling reporters, iPSC and organoid models, human tissue studies, disease-stage-specific analyses, and matched axis-level and disease-level readouts. Finally, the therapeutic section now avoids broad cross-disease claims and instead emphasizes stratified testing in models with strong genetic or mechanistic links to the axis, such as NEK1/C21orf2-linked ALS, TTBK2-linked SCA11, LRRK2-dependent PD, and HTT-HAP1-PCM1-dependent HD models.

Finally, several recent reviews already consider centrosomes and primary cilia in neurodegeneration, signal caution about over-generalization, and highlight major gaps in causal evidence. So, the ambition to cast a universal cilium/centrosome-based mechanism over the full spectrum of neurodegenerative and psychiatric disorders is not as novel as implied. For example, Lattao 2025 (https://doi.org/10.1098/rsob.240317) argues that although many disease-linked proteins localize to centrosomes or cilia, there remains little direct evidence that these organelles are central drivers of neurodegeneration. Takahashi 2025 (https://doi.org/10.3389/fnins.2025.1688839) claims that ciliary involvement in ALS remains preliminary and calls for much more rigorous validation before considering cilia as a foundational pathogenic axis. These reviews do not appear to be cited in the manuscript, whereas they treat cilia and centrosomes as possible contributors but not as a unified paradigm for neurodegeneration. That context renders the present manuscript's claims of a "new hallmark" and a universal organelle-based mechanism overly ambitious and insufficiently grounded in the existing literature.

We thank the reviewer for pointing out these relevant recent reviews. We were aware of these articles and considered them carefully while revising the manuscript, but given the breadth of the literature covered, we inadvertently omitted them from the original reference list. We have now added these citations to the revised manuscript.

Importantly, our review has a distinct scope from the articles noted by the reviewer. One focuses primarily on ciliary involvement in ALS, whereas the other discusses centrosomes in neurodegeneration more broadly. In contrast, our revised review provides a disease-by-disease synthesis across multiple NDDs, including AD, PD, ALS, HD, and hereditary ataxias, and integrates centriolar satellites as a major component of the centrosome-cilium axis. We have expanded this aspect by discussing satellite composition, regulation, function, and satellite-linked mechanisms in HD and schizophrenia.

We also agree that centrosome and cilium dysfunction should not be presented as a universal or foundational pathogenic mechanism across all NDDs. Accordingly, we removed language referring to a “unifying mechanism” or “new hallmark” and now frame centrosome-cilium-satellite dysfunction as a context-dependent contributor that intersects with established neurodegenerative pathways in defined disease and cell type contexts. The revised manuscript is therefore positioned as an evidence-based review that explicitly considers causality, disease heterogeneity, cell type specificity, and disease-stage dependence.

In summary, this work is most useful as an introduction to centrosome-cilium biology. However, it does not provide a rigorous or balanced evaluation of the wider neurodegeneration field, and its central thesis is insufficiently supported by human genetics, disease heterogeneity or mechanistic clarity. The extent of speculation and conceptual overreach means the manuscript reads more like a perspective or opinion piece advocating a unifying hypothesis than an evidence-based review. In my judgement, this makes it unsuitable for publication in EMBO reports. I do not recommend acceptance.

We appreciate the reviewer’s assessment and have revised the manuscript substantially in response. At the same time, we respectfully disagree that the manuscript is primarily speculative or only useful as an introduction to centrosome-cilium biology. The review synthesizes a broad and rapidly expanding body of literature linking centrosomes, cilia, and centriolar satellites to multiple neurodegenerative and neurodevelopmental disease contexts, and we believe that this axis represents an important emerging area for the NDD field.

We agree, however, that the original framing may have overstated the explanatory reach of this axis and may not have sufficiently distinguished established mechanisms from emerging or disease-specific observations. We have therefore revised the manuscript to make the argument more balanced, evidence-based, and clinically cautious. The revised version no longer presents centrosome-cilium-satellite dysfunction as a unifying mechanism or a new disease hallmark. Instead, it frames this axis as a context-dependent organelle-level system that intersects with established NDD mechanisms, including proteostasis failure, cytoskeletal disorganization, mitochondrial stress, impaired trafficking, and neuroinflammation.

The major revisions include:

- removal of “unifying mechanism” and “new hallmark” language;
- replacement of “complex” with “axis” where appropriate;
- stronger discussion of classical NDD hallmarks;
- addition of a ciliopathy section addressing neurodevelopmental and maintenance phenotypes;
- clearer separation of schizophrenia from classical NDDs;
- disease-specific interpretation of evidence strength and causality;
- expanded satellite-focused content;
- revised therapeutic section with concrete examples, synergy/additivity evidence, and risk-mitigation strategies;
- revised Conclusions/Future Directions centered on context, causality, cell type specificity, and disease-stage dependence;
- addition of Box 1 (“In need of answers”) summarizing major open questions for the field;
- Revised Figures 1-3, figure legends, and Table 1 legend to avoid implying uniform causality or equivalent evidence strength across diseases.

We hope that these changes address the reviewer’s concerns while preserving the central contribution of the review: an extensive, evidence-based synthesis of literature connecting centrosomes, cilia, and centriolar satellites to neurodegenerative disease mechanisms.

Referee #3:

The authors provide a revision of the current evidence identifying the cilium-centrosome-satellite axis as a possible unifying mechanisms to explain the cellular processes underlying neurodegeneration. I liked the general idea. However, that the final goal of describing the function of satellites in neurodegenerative disorders has not been reached. I found the review dispersive and not clear for what concerns the molecular aspects.

We thank the reviewer for the positive assessment of the overall concept and for identifying areas where the manuscript could be strengthened. We agree that the original version did not sufficiently develop the centriolar satellite component relative to centrosomes and cilia, and that some molecular connections required clearer organization.

In the revised manuscript, we now present centriolar satellites as established regulators of centrosome organization, ciliogenesis, ciliary trafficking, proteostasis, autophagy, and stress responses, while also acknowledging that their direct disease-specific roles in classical NDDs remain less well characterized than those of centrosomes and primary cilia. We also revised the disease sections to highlight where satellite biology is currently strongest, particularly Huntington’s disease, through the HTT-HAP1-PCM1 pathway, and schizophrenia-related neurodevelopmental models, through PCM1-, DISC1-, BBS4-, and SDCCAG8-associated mechanisms.

We further revised the manuscript to avoid presenting the centrosome-cilium-satellite axis as a single unifying mechanism for neurodegeneration. Instead, the revised review frames this axis as a context-dependent cellular system whose relevance varies across diseases, genetic contexts, cell types, and disease stages. We also revised the Conclusions/Future Directions and added Box 1 ("In need of answers") to emphasize satellite-specific open questions and readouts, including PCM1 organization, satellite positioning, satellite proteome remodeling, autophagic turnover, local translation, and aggresome-associated functions. These revisions strengthen the satellite focus of the review while placing the field's major unresolved questions in a clear experimental framework.

1. The term 'complex' typically refers to a physical assembly of proteins that interact directly to form a defined structural unit. In contrast, the centrosome-cilium-centriolar satellites system does not represent a single physical entity but rather a functional and spatial continuum. Therefore, referring to it as an 'axis' is more appropriate than complex.

We thank the reviewer for this helpful clarification. We agree that "complex" could incorrectly imply a single physical assembly of directly interacting proteins. To avoid this ambiguity, we have revised the terminology throughout the manuscript and now refer to this system as the centrosome-cilium-satellite axis. We also clarified that by "axis" we mean a spatially and functionally connected organelle network, rather than a single physical protein complex.

2. The composition of centriolar satellites should be more clearly explained, as this is fundamental to understanding their scaffolding and regulatory functions. Expanding on how centriolar satellites contribute to the regulation of ciliogenesis through autophagic pathways would strengthen the narrative and provide a more comprehensive view of their functional significance. Clarifying this interplay would help readers better understand the mechanistic connections underlying satellite-mediated control of cilia formation.

We thank the reviewer for this helpful suggestion. We have revised the centriolar satellite section to better explain satellite composition, scaffolding, and function. The revised text now clarifies that PCM1 is a core satellite scaffold and essential to satellite assembly. We also added information from quantitative mass spectrometry and proximity-labeling studies showing that satellites contain diverse set of centrosomal and ciliary proteins, microtubule-associated proteins, RNA-binding proteins, quality-control factors, and mRNAs.

We also expanded the discussion of how satellites regulate ciliogenesis through autophagy. Specifically, we now describe the cargo- and context-dependent relationship between autophagy and ciliogenesis, including selective degradation of satellite-localized OFD1, autophagy-dependent regulation of IFT20, and doryphagy-mediated turnover of PCM1-containing satellites. These additions clarify how satellites contribute to ciliary trafficking, ciliogenesis, autophagy, proteostasis, and centrosome integrity.

3. The manuscript could provide a more consistent explanation of the inheritance patterns

for all the diseases discussed. In some cases, such as the spinocerebellar ataxias (SCAs), the genetic inheritance is clearly described, whereas for other disorders the inheritance patterns are not explained in sufficient detail. Including this information throughout would improve clarity and help readers better understand the genetic basis of the diseases.

We agree that clearer and more consistent information on inheritance patterns and genetic architecture improves the reader's ability to interpret disease-specific evidence. We have revised the disease-introduction paragraphs accordingly.

In AD, we now distinguish late-onset sporadic disease from rare autosomal-dominant familial forms involving APP, PSEN1, and PSEN2. In PD, we clarify that most cases are sporadic, whereas a minority are monogenic, including autosomal-dominant forms linked to SNCA and LRRK2 and autosomal-recessive early-onset forms associated with PRKN and PINK1. In ALS, we state that most cases are sporadic, while a subset are familial, and we introduce key ALS genes including SOD1, C9orf72, TARDBP, FUS, and NEK1.

We also standardized the genetic-context statements for SCAs, HD, and schizophrenia. SCAs are described as genetically heterogeneous, usually autosomal-dominant disorders; HD as an autosomal-dominant CAG repeat expansion disorder caused by mutation in HTT; and schizophrenia as a complex polygenic neuropsychiatric disorder rather than a classical NDD. These revisions support the broader conceptual framework of the manuscript by distinguishing sporadic, familial, monogenic, and polygenic contexts when interpreting centrosome-cilium-satellite dysfunction.

4. Page 5: 'Beyond its MTOC role, the centrosome also acts as a signaling hub by concentrating regulatory molecules involved in processes cell cycle control, DNA damage response, and other pathways 16. Structurally, the centrosome consists of two centrioles surrounded by the pericentriolar material (PCM), which contains microtubule- nucleating factors such as gamma-tubulin complex that confer the centrosome's MTOC activity. Centrioles are microtubule-based cylindrical structures that are structurally and functionally distinct, with the mother centriole has distal and subdistal appendages, whereas the daughter centriole lacks them.' (This section contains several wording and grammatical issues that should be corrected for clarity. Revising these points would improve readability)

We thank the reviewer for pointing this out. We have revised this paragraph to improve grammar, readability, and scientific precision. In particular, we clarified the centrosome's signaling role, corrected the description of the pericentriolar material and γ-tubulin complex, revised the wording describing mother and daughter centrioles, and clarified the relationship between the mother centriole and basal body during ciliogenesis.

5. Motile cilia are mentioned without prior introduction or proper context within the framework of neurodegenerative diseases. Providing a brief explanation of their relevance would help complement the topic integrating with some other literature material such as Ma, X. Y., Yang, T. T., Liu, L., Peng, X. C., Qian, F., & Tang, F. R. (2023).

Ependyma in Neurodegenerative Diseases, Radiation-Induced Brain Injury and as a Therapeutic Target for Neurotrophic Factors. Biomolecules, 13(5), 754. https://doi.org/10.3390/biom13050754)

We agree that motile cilia required clearer introduction and better integration into the review. We have revised the manuscript to introduce motile cilia earlier and to explain their relevance to brain homeostasis and neurodegenerative disease mechanisms.

Specifically, in the primary cilium section, we now clarify that although the review focuses mainly on primary cilia, motile cilia of ependymal cells are also relevant because they regulate cerebrospinal fluid flow. We then expand this point in the new ciliopathy section, where we discuss how impaired ependymal motile cilia can contribute to hydrocephalus, ventricular homeostasis defects, and altered CSF circulation. We also integrated this context into the AD section, where we discuss evidence that Aβ exposure impairs ependymal ciliary beating and reduces CSF flow, linking motile cilia to AD-associated clearance biology while distinguishing this pathway from primary-cilium defects in neurons and glia. We have also added relevant literature on ependymal cells and neurodegenerative disease, including the review suggested by the reviewer.

6. It would be helpful to review the gene nomenclature throughout the text, as it is often inconsistent or incorrect.

We have reviewed gene and protein nomenclature throughout the manuscript. Human gene symbols are now formatted according to standard nomenclature conventions, whereas protein names are kept non-italicized. Species-specific nomenclature has also been standardized for mouse, zebrafish, and C. elegans genes where relevant. We also updated older or ambiguous gene names where appropriate, including replacing PARK2 with PRKN, using TARDBP for the gene encoding TDP-43, and clarifying CFAP410/C21orf2 nomenclature at first mention.

7. Page 25: 'Beyond its mitotic function, Ataxin-10 interacts with ciliary transition zone and basal body proteins, including NPHP5 and ARL13B, both critical for ciliogenesis.' (Listing ARL13B with basal body proteins may be misleading, as it primarily localizes to the ciliary membrane and axoneme. Clarification of its role in ciliogenesis is recommended.)

We revised the sentence to:

“Ataxin-10 also interacts with ciliopathy-associated proteins, including the transition-zone protein NPHP5 and the ciliary GTPase ARL13B, which are both critical for ciliogenesis.”

8. The manuscript highlights multi-target approaches to restore both ciliary and classical neurodegenerative pathways, which is a strong conceptual advance. However, the discussion remains largely descriptive. The authors should provide more concrete examples or models of combinatorial interventions, including evidence for synergy, potential risks, and strategies to mitigate off-target effects. Clarifying how these

approaches could be prioritized for preclinical or clinical testing would strengthen the translational impact of the review.

We thank the reviewer for this constructive suggestion. We have substantially revised the therapeutic section to make it more concrete and translationally focused. The revised section now distinguishes strategies aimed at restoring centrosome-cilium-satellite structure and dynamics, modulating ciliary signaling, correcting disease-linked genetic or transcript-level defects, and developing rational combinatorial approaches.

We added specific examples in defined disease contexts. For structural rescue, we now discuss HDAC6 inhibition in NEK1-linked ALS models, where HDAC6 inhibition restores ciliary defects in patient-derived cells and iPSC-derived motor neurons. For signaling modulation, we expanded the discussion of Shh and TGF-β pathways to emphasize that therapeutic activation or inhibition must be tailored to pathway state, cell type, and disease stage. We also discuss LRRK2-dependent PD models as an example in which LRRK2 kinase inhibition can restore Rab-dependent ciliary trafficking defects and should be evaluated together with lysosomal, mitochondrial, vesicular-trafficking, dopaminergic, and motor readouts.

To address combinatorial evidence, we added examples from ciliopathy models. In a polycystic liver disease model, combined HDAC6 inhibition with ACY-1215 and somatostatin receptor modulation with pasireotide synergistically reduced cyst growth. We also added an ADPKD example in which tolvaptan combined with pasireotide produced an additive reduction in cyst progression. We explicitly state that these studies support the feasibility of rational combinatorial design in ciliopathy models, but should not be interpreted as direct evidence for NDD therapy without validation in neuronal and glial systems.

We also added a prioritization framework for future testing. We now propose that combinations should be evaluated first in models with strong genetic or mechanistic links to the axis, such as NEK1/C21orf2-linked ALS, TTBK2-linked SCA11, LRRK2-dependent PD, and HTT-HAP1-PCM1-dependent HD models. We further emphasize that efficacy should be assessed using matched axis-level and disease-level readouts, and that risk mitigation should include dose-response studies, cell type-specific assays, disease-stage-aware intervention windows, assessment of ciliary and non-ciliary effects, and safety monitoring in tissues where the targets have known physiological roles.

Finally, we revised Figure 3 and its legend to reflect this more cautious translational framework. The figure now presents therapeutic strategies as potential preclinical entry points rather than established disease-modifying therapies for NDDs. onsistent with this revision, Box 1 now highlights therapeutic stratification as a major open question and emphasizes the need to pair ciliary, centrosomal, and satellite readouts with disease-relevant functional outcomes.

9. There are several elements in Figure 1 that need revision. In panel A, the arrow connecting Dynein-2 to the structure is projected onto the axoneme rather than the protein

itself. Additionally, placing PCM1 in parentheses may be misleading, as it suggests that the structure consists solely of this protein. It would be more appropriate to indicate the Distal Appendages and Sub-distal Appendages in panel A rather than panel B. In panel B, near number 1, the disc that initiates recruitment of the proteins responsible for blocking elongation is not indicated. Finally, points 4 and 5 could be depicted more clearly to better illustrate the disassembly process.

We thank the reviewer for these helpful suggestions. We have revised Figure 1 accordingly. In panel B, we added the CP110-CEP97 cap at the distal end of the mother centriole to show the structure that suppresses premature axoneme extension. The revised figure and legend now clarify that pre-ciliary vesicles are recruited while the CP110-CEP97 cap is present, and that TTBK2 recruitment promotes CP110-CEP97 cap removal and IFT recruitment before axoneme initiation. We also revised the later steps to depict cilium disassembly more clearly, including ciliary resorption and katanin-dependent scission. We also revised the Figure 1 legend to improve accuracy and terminology. The revised legend now describes the centrosome-cilium-satellite axis, PCM1-scaffolded centriolar satellites, distal and subdistal appendages, IFT-mediated transport, transition-zone function, and the timing of CP110-CEP97 cap removal during ciliogenesis.

In addition, we updated Figures 2 and 3 and the Table 1 legend to align with the revised framework of the manuscript. Figure 2 now presents disease-associated findings as context-dependent centrosome-cilium-satellite axis alterations, and its legend clarifies that the examples do not imply equivalent causal strength across diseases. Figure 3 now frames therapeutic strategies as potential preclinical entry points that require validation with both axis-level and disease-relevant readouts. We also revised the Table 1 legend to describe the entries as reported intersections between disease-associated pathways and centrosomes, basal bodies, primary cilia, motile cilia, or centriolar satellites.

Prof. Elif Firat-Karalar
Koc University
Molecular Biology and Genetics
Rumeli Feneri Yolu
Istanbul 34450
Turkey

Dear Prof. Firat-Karalar,

I am pleased to inform you that your manuscript has been accepted for publication in EMBO reports. Your manuscript will be processed for publication by EMBO Press. It will be copy edited and you will receive page proofs prior to publication. Please note that you will be contacted by Springer Nature Author Services to complete licensing information.

Should you be planning a Press Release on your article, please notify the production team via this form in order to coordinate publication and release dates: https://forms.cloud.microsoft/e/eH4A8JVP6g

If you have any questions, please do not hesitate to contact the Editorial Office. Thank you for your contribution to EMBO Reports.

Yours sincerely,

Kurt Weir
Editor
EMBO Reports

>>> Please note that it is EMBO Reports policy for the transcript of the editorial process (containing referee reports and your response letter) to be published as an online supplement to each paper. If you do NOT want this, you will need to inform the Editorial Office via email immediately. More information is available here: https://link.springer.com/partners/embo-press/editorial-policies#Peer%20review
